# Time course of lung retention and toxicity of inhaled particles: short-term exposure to nano-Ceria

**DOI:** 10.1007/s00204-014-1349-9

**Published:** 2014-10-02

**Authors:** Jana Keller, Wendel Wohlleben, Lan Ma-Hock, Volker Strauss, Sibylle Gröters, Karin Küttler, Karin Wiench, Christiane Herden, Günter Oberdörster, Bennard van Ravenzwaay, Robert Landsiedel

**Affiliations:** 1 Experimental Toxicology and Ecology, BASF SE, 67056 Ludwigshafen am Rhein, Germany; 2 Material Physics, BASF SE, Ludwigshafen am Rhein, Germany; 3 Product Safety, BASF SE, Ludwigshafen am Rhein, Germany; 4 Institute of Veterinary Pathology, Justus-Liebig-University, Giessen, Germany; 5 Department of Environmental Medicine, University of Rochester, Rochester, NY USA

**Keywords:** Nanomaterial (NM), Inhalation, Ceria, Respiratory tract, Bronchoalveolar lavage

## Abstract

Two Ceria nanomaterials (NM-211 and NM-212) were tested for inhalation toxicity and organ burdens in order to design a chronic and carcinogenicity inhalation study (OECD TG No. 453). Rats inhaled aerosol concentrations of 0.5, 5, and 25 mg/m^3^ by whole-body exposure for 6 h/day on 5 consecutive days for 1 or 4 weeks with a post-exposure period of 24 or 129 days, respectively. Lungs were examined by bronchoalveolar lavage and histopathology. Inhaled Ceria is deposited in the lung and cleared with a half-time of 40 days; at aerosol concentrations higher than 0.5 mg/m^3^, this clearance was impaired resulting in a half-time above 200 days (25 mg/m^3^). After 5 days, Ceria (>0.5 mg/m^3^) induced an early inflammatory reaction by increases of neutrophils in the lung which decreased with time, with sustained exposure, and also after the exposure was terminated (during the post-exposure period). The neutrophil number observed in bronchoalveolar lavage fluid (BALF) was decreasing and supplemented by mononuclear cells, especially macrophages which were visible in histopathology but not in BALF. Further progression to granulomatous inflammation was observed 4 weeks post-exposure. The surface area of the particles provided a dose metrics with the best correlation of the two Ceria’s inflammatory responses; hence, the inflammation appears to be directed by the particle surface rather than mass or volume in the lung. Observing the time course of lung burden and inflammation, it appears that the dose rate of particle deposition drove an initial inflammatory reaction by neutrophils. The later phase (after 4 weeks) was dominated by mononuclear cells, especially macrophages. The progression toward the subsequent granulomatous reaction was driven by the duration and amount of the particles in the lung. The further progression of the biological response will be determined in the ongoing long-term study.

**Electronic supplementary material:**

The online version of this article (doi:10.1007/s00204-014-1349-9) contains supplementary material, which is available to authorized users.

## Introduction

During the production and processing of nanomaterials in the workplace, releases into the air may occur (Kuhlbusch et al. 2011). Therefore, inhalation is considered to be the major route of concern. Furthermore, pulmonary inflammation, fibrosis, and lung cancer are most important adverse effects after chronic inhalation exposure of rats to poorly soluble, non-fibrous (nano)particles of low toxicity (PSP) (Oberdorster 2002). Tumor formation in rat lungs after long-term PSP exposure is thought to be caused by altered particle clearance kinetics involving chronic inflammation associated with oxidative stress, secondary genotoxicity, and cell proliferation (Greim and Ziegler-Skylakakis 2007; ILSI Risk Science Institute Workshop Participants 2000). A previous study observed that excessive amounts of the microscale Titania induced lung tumors in rats after 2-year inhalation exposure to a high concentration of 250 mg/m^3^ (Lee et al. 1985). Also, nanoscale materials, Titania (P25) and carbon black (Printex 90), caused lung tumors in rats after 2 years of inhalation to 10 and 11.7 mg/m^3^, respectively (Heinrich et al. 1995; Nikula et al. 1995). The tumor incidence observed in these studies was high (32–29 %). Only high concentrations were tested in these studies, and no information about detailed deposition, clearance, and retention was provided. Thus, the existing studies contribute little to the understanding of the mechanisms of nanoparticle-induced tumor formation by long-term inhalation (Becker et al. 2011). Standardized long-term studies according to the test guidelines with a distinct study design and concentration selection focusing on exposure dose–response relationships are crucial for risk assessment and derivation of occupational safety values, as well as for classification and labeling under REACH (Becker et al. 2011; European Commission 2013).

Therefore, an appropriate long-term inhalation study has been initiated as a collaboration of the German government with BASF within the OECD sponsorship programme and the EU-project NanoREG (Teunenbroek et al. 2013; Federal Ministry for the Environment 2012). This study is performed according to OECD test guideline No. 453 (OECD 2009a). Nano-Ceria was selected as a test material representing a poorly soluble particle because of its importance for industrial applications. Nano-Ceria is used as an UV-absorbent, a polishing agent for silicon wafers, and a fuel additive to decrease diesel particle emission (Cargnello et al. 2013). The OECD has added Ceria to the list of representative manufactured nanomaterials for toxicological evaluations (NM-211, NM-212, NM-213) (Demokritou et al. 2012; OECD 2010). The short-term studies with 5 days and 4 weeks of exposure presented in this manuscript provide information on biokinetics and effects of nano-Ceria required for the design of a long-term inhalation study.

Till now, the available knowledge concerning inhalation toxicity of Ceria is limited. In an acute study, 4-h nose-only exposure to 641 mg/m^3^ nano-Ceria in rats resulted in pulmonary inflammation and the formation of multifocal microgranulomas in lung after 14 days of post-exposure. This was explained by the impaired clearance of the particles from the lung (Srinivas et al. 2011). In a whole-body 4-day inhalation study, exposing rats to 2.7 mg/m^3^ nano-Ceria 2 h/day, lung injury, and inflammation were observed after 1 day post-exposure (Demokritou et al. 2012). In a 28-day inhalation study of micro- and nano-Ceria (10.79 mg/m^3^ NM-211, 19.95 mg/m^3^ NM-212, 55.0 mg/m^3^ NM-213) in rats, high lung burdens, distribution of inhaled Ceria to various extrapulmonary organs, and slow tissue clearance from the lung were observed (Geraets et al. 2012). Furthermore, 28-day nose-only inhalation of 2 mg/m^3^ nano-Ceria in mice induced severe and chronic inflammation including necrosis; proteinosis; fibrosis and granulomas in the lungs; and severe changes in kidney, liver, and heart (Aalapati et al. 2014). These strong effects were associated with significant accumulation of the nanoparticles in the (extra)pulmonary tissues and indicated that inhalation of Ceria can lead to pulmonary and extrapulmonary toxicity. There were also a few instillation studies assessing toxicity of nano-Ceria (He et al. 2010; Nalabotu et al. 2011; Ma et al. 2011, 2014; Molina et al. 2014). After single intratracheal instillation of 0.15, 0.5, 1.3, 5, and 7 mg/kg in male Sprague–Dawley rats, Ceria caused concentration-dependent alveolar macrophage functional change, significant lung inflammation, and cytotoxicity, indicating a potential shift from a proinflammatory environment to final pulmonary fibrosis (Ma et al. 2011). However, instillation studies usually have a bolus of high dose and high dose rate in the lung and are therefore less suitable to determine the exposure concentrations, biokinetics, and biological effects toward the design of a long-term inhalation study (Baisch et al. 2014). The majority of the published studies indicate an inflammatory potential of Ceria but lack appropriate dose metrics and biokinetic information (Cassee et al. 2011; Becker et al. 2011).

The short-term studies with 5 days and 4 weeks of exposure described in this manuscript provide data on biokinetics as well as pulmonary and potential extrapulmonary effects of two nano-Ceria (NM-211 and NM-212) using established and standardized study protocols. Whole-body exposure is the only option for exposure routes in long-term studies for animal welfare reasons because the animals are not restrained during the exposure. Furthermore, oral and dermal uptake of different micro- and nano-sized test materials has been proven to be negligible (Landsiedel et al. 2012; Gamer et al. 2006; Pflucker et al. 2001; Molina et al. 2014).

Physicochemical properties of nanoparticles can have a significant influence on their fate and biological effects (Oberdorster et al. 1994a; Anjilvel and Asgharian 1995). Therefore, the two nanoparticles were characterized in accordance with the nano-specific guidance in REACH, as validated earlier with other materials from the OECD sponsorship programme (Wohlleben et al. 2013). Persistence and solubility in different media were investigated to study the bioavailability of the tested Ceria. Comprehensive characterizations of the test atmospheres were performed according to the respective guidelines and OECD guidance documents (ECHA 2012; Hussain et al. 2009).

## Materials and methods

### General

Two short-term studies (5 days and 4 weeks of exposure) were designed using two different nano-Ceria. In the short-term study with 5 days of exposure, we compared two Ceria (NM-212 and NM-211) with regard to their inhalation toxicity, deposition, and clearance kinetics. Ceria (NM-212) was tested further after 4 weeks of exposure. In order to compare the effects after different exposure durations, same target concentrations were selected for the two studies. Biological effects of Ceria were studied by the analysis of bronchoalveolar lavage fluid (BALF) and blood and histopathology of the respiratory tract. Biokinetics were assessed by the determination of lung and lung-associated lymph node burdens at different time points. The short-term inhalation studies were performed according to the OECD Principles of Good Laboratory Practice (GLP) [Organization for Economic Cooperation and Development (OECD) 1998]. The study design of the study with 4 weeks of exposure was carried out according to the OECD guidelines for testing of chemicals, Section 4: Health Effects, No. 412, with additional modifications [Organization for Economic Cooperation and Development (OECD) 2009b]. To increase the comparability, the short-term studies were performed in the same inhalation laboratory under the same test conditions, determining same end points for assessing biological effects and deposition and clearance kinetics. This facilitated a direct comparison between the measured parameters.

### Test materials and characterization

The two Ceria test materials in this work are NM-211 and NM-212 received from the OECD sponsorship programme for safety testing of manufactured nanomaterials (Hussain et al. 2009). Very recently, the European Chemicals Agency (ECHA) drafted a nano-specific guidance document, designated as Appendix R7-1 (Hermans 1963). The following end points for the characterization of the aerosol materials were considered as follows: agglomeration/aggregation, particle size distribution, water solubility/dispersability, crystalline phase and size, representative electron microscopy (TEM) for morphology, specific surface area, zeta-potential (surface charge), surface chemistry, photocatalytic activity, purity and impurities, and porosity. The methods to characterize these properties are described in detail in a previous work (Wohlleben et al. 2013). The agglomerate density of Ceria NM-212 was derived from mercury porosimetry (see Fig. S1). By integrating all void volumes up to the agglomerate outer dimensions of 1.1 µm, the agglomerate density of NM-212 would be 2.0 g/m^3^ based on the mercury porosimetry measurement. The density of NM-211 was determined consistently. The agglomerate density was used in the calculation of expected lung burden by multiple-path particle dosimetry (MPPD) model (Anjilvel and Asgharian 1995).

### Animals

This study was approved by the local authorizing agency for animal experiments (Landesuntersuchungsamt Koblenz, Germany) as referenced by the approval number G 12-3-028. Animals were housed in an AAALAC-accredited facility in accordance with the German Animal Welfare Act and the effective European Council Directive. Female Wistar rats [strain: Crl:WI(Han)] were obtained at an age of 7 weeks from Charles River Laboratories (Sulzfeld, Germany). The animals were maintained in groups up to five animals in a polysulfone cage [H-Temp (PSU)], TECNIPLAST, Germany) with a floor area of about 2,065 cm^2^ (610 × 435 × 215 mm) with access to wooden gnawing blocks, GLP-certified feed (Kliba laboratory diet, Provimi Kliba SA, Kaiseraugst, Basel Switzerland), and water ad libitum. Animal room was kept at 20–24 °C and relative humidity 30–70 % with 15 air changes/h. A light/dark cycle of 12 h each was kept throughout the study periods. To adapt to the exposure conditions, the animals were acclimatized to fresh air under the study flow conditions in whole-body inhalation chambers for 2 days before the start of the exposure period. Up to 2 animals/cage were exposed in wire cages, type DKIII (BECKER & Co., Castrop-Rauxel, Germany) in a whole-body chamber. During the exposure, feed and drinking water were withdrawn from the animals.

### Inhalation system

The animals were exposed in wire cages that were located in a stainless steel whole-body inhalation chamber (*V* = 2.8 m^3^ or *V* = 1.4 m^3^). The inhalation atmospheres were passed into the inhalation chambers with the supply air and were removed by an exhaust air system with 20 air changes/h. For the control animals, the exhaust air system was adjusted in such a way that the amount of exhaust air was lower than the filtered clean, supply air (positive pressure) to ensure that no laboratory room air reaches the control animals. For the treated animals, the amount of exhaust air was higher than the supply air (negative pressure) to prevent the contamination of the laboratory as a result of potential leakages from the inhalation chambers.

### Aerosol generation and monitoring

Ceria aerosols were produced by dry dispersion of powder pellets with a brush dust generator (developed by the Technical University of Karlsruhe in cooperation with BASF, Germany) using compressed air (1.5 m^3^/h). The so generated dust aerosol was diluted by conditioned air (54.5 m^3^/h) passed into whole-body inhalation chambers. The control group was exposed to conditioned, clean air. The desired concentrations were achieved by varying the feeding speed of the substance pellet and by varying the rotating speed of the brush. Based on the data of a comprehensive technical trial, the aerosol concentrations within the chambers were considered to be homogenous (data not shown). Nevertheless, the positions of the exposure cages were rotated within each chamber. Generated aerosols were continuously monitored by scattered light photometers (VisGuard, Sigrist). Particle concentrations in the inhalation atmospheres were analyzed by gravimetric measurement of air filter samples. Particle size distribution was determined gravimetrically by cascade impactor analysis using eight stages Marple personal cascade impactor (Sierra Anderson, USA). In addition, light-scattering aerosol spectrometer (WELAS^®^ 2000, Palas, Karlsruhe, Germany) was used to measure particles from 0.24 to 10 µm. To measure particles in the submicrometer range, scanning mobility particle sizer (SMPS 5.400, Grimm Aerosoltechnik, Ainring, Germany) was used. The sampling procedures and measurements to characterize the generated aerosols were described in detail in a previous work (Ma-Hock et al. 2007).

### Study design of short-term studies (5 days and 4 weeks of exposure)

**Fig. 1:**
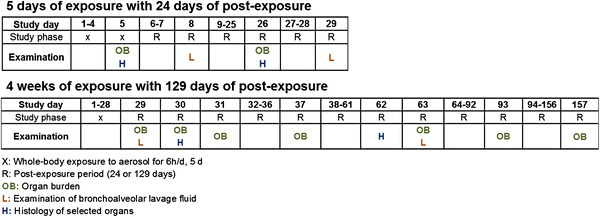
Study design of short-term studies with 5 days and 4 weeks of exposure

In general, the animals were whole-body exposed to dust aerosols for 6 h/day on 5 consecutive days/week with a respective post-exposure period (see Fig. 1). The highest aerosol concentration was 25 mg/m^3^, which was expected to cause biological effects and should lead to lung overload at least for 20 exposures. The mid and low aerosol concentrations were 5 and 0.5 mg/m^3^. The low aerosol concentration with an expected lung burden far below the overload condition should not lead to any adverse effects. The mid aerosol concentration, which was spaced tenfold higher than the low concentration, was expected to cause some biological effects. The post-exposure period and the examination time points were scheduled to address the progression or regression of the biological effects, with their correlation to lung burden and lung clearance kinetics.

### Clinical examinations

Clinical observations of the animals were recorded for each animal at least three times per day on exposure days and once a day during the pre-exposure and post-exposure periods. Signs and findings were recorded for each animal. During exposure, examination was possible only on a group basis. The animals were weighed prior to the pre-exposure period, at the start of the exposure period (day 0), and twice weekly until killing or twice within the 5 exposure days.

### Hematology and clinical chemistry

Blood sampling of five fasted rats per test group was performed in the morning by retro-orbital venous plexus puncture under isoflurane anesthesia (Isoba^®^, Essex GmbH Munich, Germany). The extent of the examination was according to the data requirements of OECD test guideline 412. The parameters such as red blood cell counts, hemoglobin, hematocrit, mean corpuscular volume (MCV), mean corpuscular hemoglobin content (MCH), mean corpuscular hemoglobin concentration (MCHC), platelet counts, total white blood cell as well as differential blood cell counts were determined in the EDTA-stabilized blood with a hematology analyzer (ADVIA^®^ 120, Siemens Diagnostics, Fernwald, Germany). Additionally, acute-phase proteins were determined in serum: rat haptoglobin and rat γ2-macroglobulin (ELISA by Immunology Consultants Laboratory Inc., Newberg, OR, USA) were measured with a Sunrise MTP Reader (Tecan AG, Switzerland) by using the Magellan Software provided by the instrument producer. The enzyme levels [alanine aminotransferase (ALT), aspartate aminotransferase (AST), alkaline phosphatase (ALP), γ-glutamyltransferase (GGT)] and different blood parameters of clinical chemistry were evaluated by using an automatic analyzer (Hitachi 917; Roche, Mannheim, Germany).

### Systemic genotoxicity (micronucleus test)

Systemic genotoxicity (micronucleus test) was performed in the short-term studies with 5 days and 4 weeks of exposure (3 and 2 days after the end of exposure, respectively). Micronucleus analysis in erythrocytes of rat peripheral blood was performed with the MicroFlow^®^ plus (Rat Blood) kit from Litron Laboratories, Rochester, NY, USA. The flow cytometric differentiation of reticulocytes as well as normochromic erythrocytes with and without micronuclei was done on the FacsCalibur instrument (BectonDickinson, Heidelberg, Germany) after staining of the cells with CD71 and propidium iodide.

### Bronchoalveolar lavage

Five animals per test group were killed by exsanguination from the aorta abdominals and vena cava under pentobarbital (Narcoren^®^) anesthesia. The lungs of the animals were lavaged in situ twice with 6 mL (22 mL/kg body weight) of 9 % (w/v) saline solution. A total of 11 mL BALF was obtained per animal for analysis. Aliquots of the BALF were used for the determinations of total protein concentration, total cell count, differential cell count, and activity of the enzymes. In the short-term study with 5 days of exposure, BALF was analyzed 3 and 24 days after the end of exposure. After 4 weeks of exposure, lavaged lungs and aliquots of the BALF (1 mL) were stored at −80 °C and used for the determination of the Cer content in the lung (lung burden) 1 and 35 days after the end of exposure. Total BALF cell counts were determined with an ADVIA^®^ 120 (Siemens Diagnostics, Fernwald, Germany) hematology analyzer. Counts of macrophages, polymorphonuclear neutrophils (PMN), lymphocytes, eosinophils, monocytes, and atypical cells were performed on Wright-stained cytocentrifuge slide preparations (Warheit and Hartsky 1993). The differential cell count was evaluated manually by counting at least 400 BALF cells per sample. Using a Hitachi 917 (Roche Diagnostics, Mannheim, Germany) reaction rate analyzer, levels of BALF total protein and activities of lactate dehydrogenase (LDH), alkaline phosphatase (ALP), γ-glutamyltransferase (GGT), and N-acetyl-β-glucosaminidase (NAG) were measured.

### Cytokines and chemokines (in BALF and serum)

Cytokines and chemokines in BALF and serum were measured at Rules-based Medicine Inc., Austin, TX, USA, with xMAP technology (Luminex Corp., Austin, TX, USA). The measured parameters comprised various cytokines, chemokines, adhesion molecules, matrix metalloproteinases, acute-phase proteins, signal proteins of apoptosis, or cell proliferation. Briefly, a list of parameters was described previously (Ma-Hock et al. 2009). After evaluation of the most sensitive parameters or reaction patterns in previous studies, the following standard parameters were chosen for characterizing the lung inflammation in BALF:

- Rat monocyte chemoattractant protein-1 level (*rat MCP*-*1*; Instant ELISA, Bender MedSystems, Vienna, Austria (cat. no BMS631INST);
- Rat cytokine-induced polymorphonuclear neutrophil chemoattractant-1 level (*rat CINC*-*1* **/** *IL*-*8*; ELISA, R&D Systems Inc., Minneapolis, USA (Quantikine rat CINC-1, cat. no. RCN100);
- Macrophage colony-stimulating factor (*M*-*CSF*; Quantikine Mouse M-CSF ELISA, R&D Systems Inc., Minneapolis, USA (cat no. MMC00);
- *Rodent osteopontin*; ELISA, R&D Systems, Inc., Minneapolis, US (Quantikine mouse osteopontin, cat. no. MOST00).

The cell mediators were measured at a sunrise MTP reader (Tecan AG, Switzerland) by using the Magellan Software provided by the instrument producer. Four chemokines and cytokines were measured using ELISA test kits: MCP-1, IL-8/CINC-1, M-CSF, and osteopontin. The detailed procedure was described previously (Ma-Hock et al. 2009).

### Pathology

In the short-term studies, necropsy and histopathology were performed after 5 days of exposure and 21 days after the end of exposure (5 days of exposure) and 2 and 34 days after the end of exposure (4 weeks of exposure). In general, five animals per test group were investigated for pathological examination in both short-term studies. For the short-term study with 4 weeks of exposure, however, ten animals were examined for pathological examination of the respiratory tract and all gross lesions. At necropsy, animals were exsanguinated by opening of the abdominal great vessels under deep pentobarbital anesthesia. All organs were preserved according to OECD TG No. 412. Following organs were weighed: adrenal glands, brain, heart, ovaries, uterus with cervix, kidney, liver, lungs, spleen, thymus, and thyroid glands. The lungs were instilled (30 cm water column) with and fixed in 10 % neutral-buffered formalin (NBF). After 24- to 48-h fixation in 10 % NBF, the lungs were transferred to 70 % ethanol. All other organs were fixed in 10 % NBF. All the organs and tissues described in the OECD TG No. 412 were trimmed according to the RITA trimming guides for inhalation studies (Kittel et al. 2004; Ruehl-Fehlert et al. 2003). After paraplast-embedding, the blocks were cut at 2- to 3-µm thickness, mounted on glass slides and stained with hematoxylin and eosin. Extrapulmonary organs and the respiratory tract compromising nasal cavity (four levels), larynx (three levels), trachea (transverse and longitudinal with carina), lung (five lobes), and mediastinal and tracheobronchial lymph nodes were assessed by light microscopy. For the lungs, whole histopathological examination was performed in animals of all test groups. For all other tissues, only the animals of the control and high concentration group of Ceria were initially examined. When changes were observed in the high concentration group, respective organs and tissues of the animals exposed to low and intermediate aerosol concentrations were also examined by light microscopy. All histopathological examinations were performed by a well-experienced board-certified veterinarian toxicopathologist followed by an internal pathology peer review.

### Organ burden analysis

Lung burden of the two different Ceria was evaluated twice, immediately after 5 days of exposure and after 21 days after the exposure end. In the short-term study with 4 weeks of exposure, Cerium content was determined at seven time points over 129 days of post-exposure period. Cerium content in the lungs, lung-associated lymph nodes, and liver of either three or five animals per test group were examined. 1 and 35 days after the end of exposure (4 weeks of exposure), the lavaged lungs and aliquots of BALF of five animals per group were used for the determination of lung burden. This examination method has likely caused a loss of the test material during preparation and handling of the lungs. Furthermore, lung burdens were measured 2 days after the end of exposure using the left half lungs of five animals/test group, only. On the basis of the availability of total lung weights, lung burdens were calculated up from the half lung burden values with the corresponding weight of the half lungs. Lung burden of the remaining time points was determined using the whole (not lavaged) lung. After digestion with mixed acid, samples of each lung or lymph node were dissolved in sulfuric acid and ammonium sulfate. ^140^Cer content in the obtained solution was analyzed by inductively coupled plasma mass spectrometry (ICP-MS, Agilent 7500 C) or by inductively coupled plasma optical emission spectrometry (ICP-OES, Varian 720-ES) with a wavelength of 419 nm. With this method, limit of detection for Cer is 0.3 μg. The amounts of Ceria in the respective tissues were calculated by measuring elemental Cerium with ICP-MS.

### Statistical analysis

For body weight changes, Dunnett’s test was used for a comparison of each test group with the control group test (Dunnett 1955; Dudewicz et al. 1975). Clinical pathology parameters (BALF cytology, enzyme data, and BALF and serum cell mediator data) were analyzed by nonparametric one-way analysis using the Kruskal–Wallis test (two-sided). If the resulting *p* value was ≤0.05, a pair-wise comparison of each test group with the control group was performed using the Wilcoxon test or the Mann–Whitney *U* test (both two-sided) (*p* ≤ 0.05 for statistical significance). Comparison of organ weights among test groups was performed by nonparametric one-way analysis using the two-sided Kruskal–Wallis test, followed by a two-sided Wilcoxon test for the hypothesis of equal medians in case of *p* ≤ 0.05.

## Results

### Characterization of test material

**Fig. 2:**
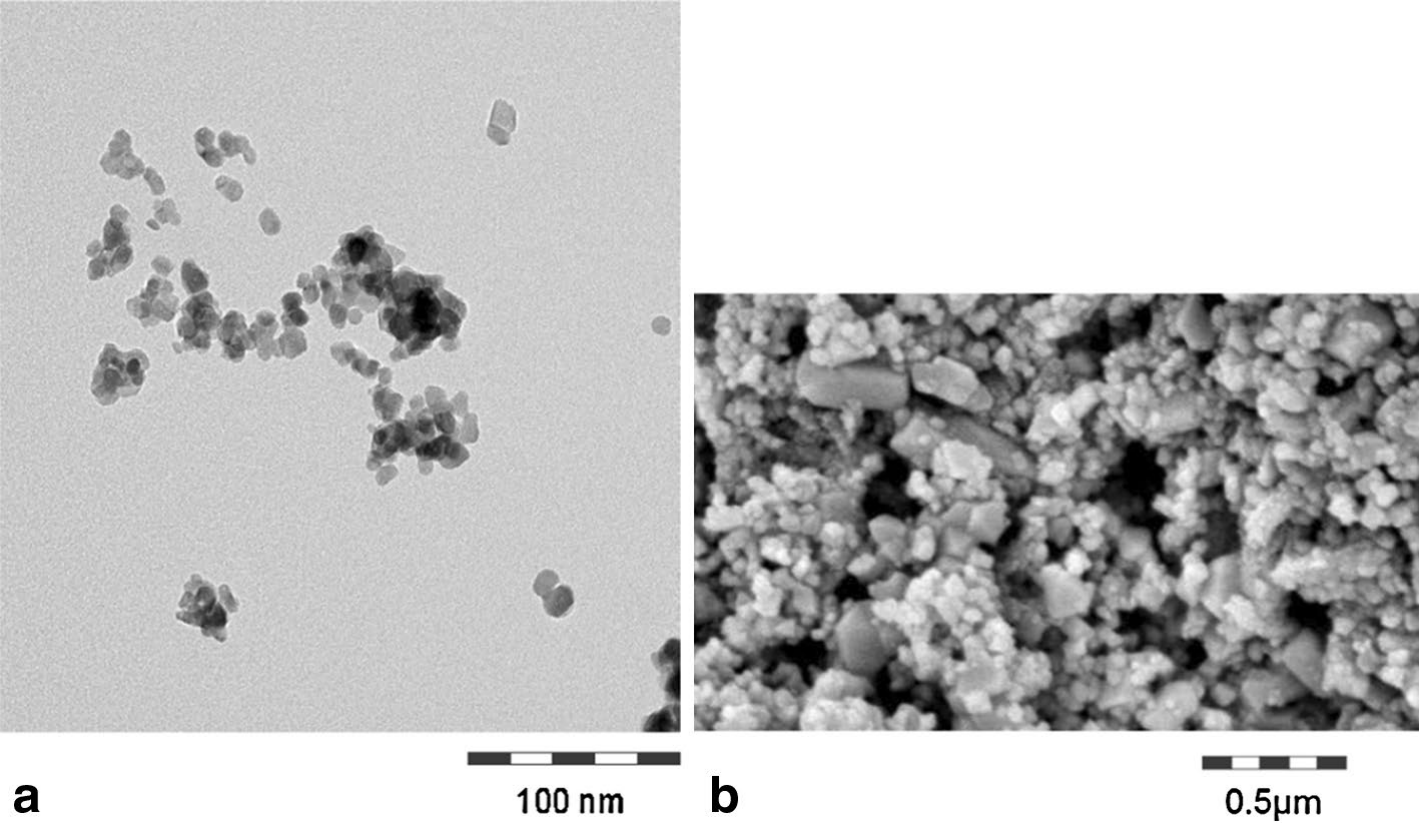
Structure of Ceria. Electron microscopy images of a representative ensemble of particles of Ceria NM-211 (**a**) and NM-212 (**b**) (see Table 1 for size characterization by complementary methods)

Both Ceria were yellowish white powders that were produced by precipitation and were nominally uncoated. We completely re-characterized the Ceria NM-211 and NM-212 based on the nano-specific guidance on physical–chemical properties (Wohlleben et al. 2013). Table 1 indicates the techniques chosen for each end point (see “Materials and methods” section) and summarizes the results. The Ceria NM-212 material had an average primary particle diameter of 40 nm, in excellent accordance of electron microscopy with the crystallite size derived from the diffraction peak width and with the sphere-equivalent diameter derived from the BET-specific surface area of 27 m^2^/g (see Fig. 2). The Ceria NM-211 material consisted of considerably smaller primary particles with number-based median diameter of 8.2 nm (from TEM) and correspondingly larger specific surface (53 m^2^/g, from BET). Primary particles of both materials were crystalline with cubic lattice as it is the characteristic for cerianite and had irregular, but roughly globular shapes. In the as-produced powder, porosimetry by Hg intrusion indicated that these particles were aggregated and agglomerated to sizes that range from a few hundred nm to tens of µm.

**Table 1:** Physical–chemical characterization of Ceria NM-212 and NM-211

OECD end points	Ceria NM-212	Ceria NM-211
Particle size distribution (TEM: primary particle diameter)/state of agglomeration (SEM: agglomerate diameter)	40 nm SEM: 3,000–150,000 nm Hg pore sizes: 35 nm, around 7 µm	4–15 nm D50: 8.2 nm Hg pore sizes: 5, 70 nm, around 10 µm
Crystallite size (XRD)	40.0 nm	12.5 nm
Crystallite phase (XRD)	Cerianite, Ceria-cubic	Cerianite, Ceria-cubic
Specific surface area (Hg, BET)	30 m^2^/g (Hg), 27 m^2^/g (BET)	33 m^2^/g (Hg), 53 m^2^/g (BET)
Surface chemistry (XPS) Atom percent	C 79.9 (C–C 62.6; C–O 7.0; C=O 3.5; COOH 6.9) O 17.7 Ce 2.4 With oxidation state Ce(III) 14 %, Ce(IV) 86 %	Ce 28.7 O 57.2 C 14.1 With oxidation state Ce(III) 22 %, Ce(IV) 78 %
Surface charge Isoelectric point = IEP ζ-Pot at pH 7 from electrophoretic mobility	IEP: >pH 10 (always cationic) +42 mV	IEP = pH 8.3 +16 mV
Photocatalytic activity		
Photon efficiency (methylene blue)	0.01 ± 0.005	0.0005 ± 0.0002
Dispersability		
D50 and average agglom. number (centrifugation)	D50 = 432 nm/AAN = 11(in water)	D50 = 2,839 nm/AAN = 346 (in water)
Solubility (ICP-MS)		
In water	0.002 wt%	<0.001 wt%
In DMEM/FCS	<0.001 wt%	<0.001 wt% (recrystallizes)
In PSF	<0.001 wt% (recrystallizes)	<0.001 wt%
In PBS	<0.001 wt%	
In FassiF	<0.001 wt%	
In 0.1 N HCl	0.02 wt% (ripening)	
Impurities (TGA, XPS)	Total content of 0.7 % organic contaminations, identified as ester + alkyl groups, found mostly on the particle surface	Total content of 1.6 % contaminations, thereof small amounts of alkyls found on the particle surface

The surface of Ceria NM-212 beared organic contaminations. An assessment by thermogravimetry (TGA) confirmed that the total organic content remained below 0.7 %, but the photoelectron signal of XPS, which had an information depth between 3 and 10 nm, indicated 80 % carbon atoms on the surface. The contamination was hence a very thin and homogeneous layer around the purely inorganic particles. According to the fit of photoelectron energies from C(1s) level and from O(1s) level, the contamination could be an ester with a long alkyl chain. In contrast, the Ceria NM-211 had only 14 % carbon atoms on the surface (by XPS), and the even more sensitive SIMS techniques confirmed that Ceria dominated the surface, with vanishing amounts of nitrates and alkyls. The Cerium atoms at crystalline edges were known to be redox-active, which was confirmed by the detection of 14 % of the Cerium as Ce(III) (within the XPS-accessible surface layer) in Ceria NM-212, respectively, 22 % for the Ceria NM-211 with its decreased radius of curvature.

If dispersed in clean water, the Ceria NM-212 surface was positively charged across the entire physiological pH range, with a zeta-potential of +42 mV at pH 7. The surface of Ceria NM-212 was rather reactive in a photocatalytic assay with a photon efficiency of 1.3 × 10^−2^, which was 5 times higher than the well-known Titania P25 (NM-105) and an order of magnitude higher than for the Ceria NM-211, leaving the possibility of the organic contaminations being responsible for that catalytic activity, observed only under UV irradiation (Wohlleben et al. 2013; Molina et al. 2014). After dispersion in water by stirring only, the dispersability was limited with average agglomeration numbers (AAN = the ratio of agglomerate diameter to primary particle diameter) above 10, based on the measurement of the resulting agglomerates that were just below the micron range (by analytical ultracentrifugation), and tended to be larger for the Ceria NM-211 than for NM-212, confirming earlier findings of the PROSPECT consortium and the European Commission’s JRC report (Hermans 1963; PROSPEcT: Ecotoxicology Test Protocols for Representative Nanomaterials in Support of the OECDSponsorship Programme 10 A.D.; European Commission 2014).

**Fig. 3:**
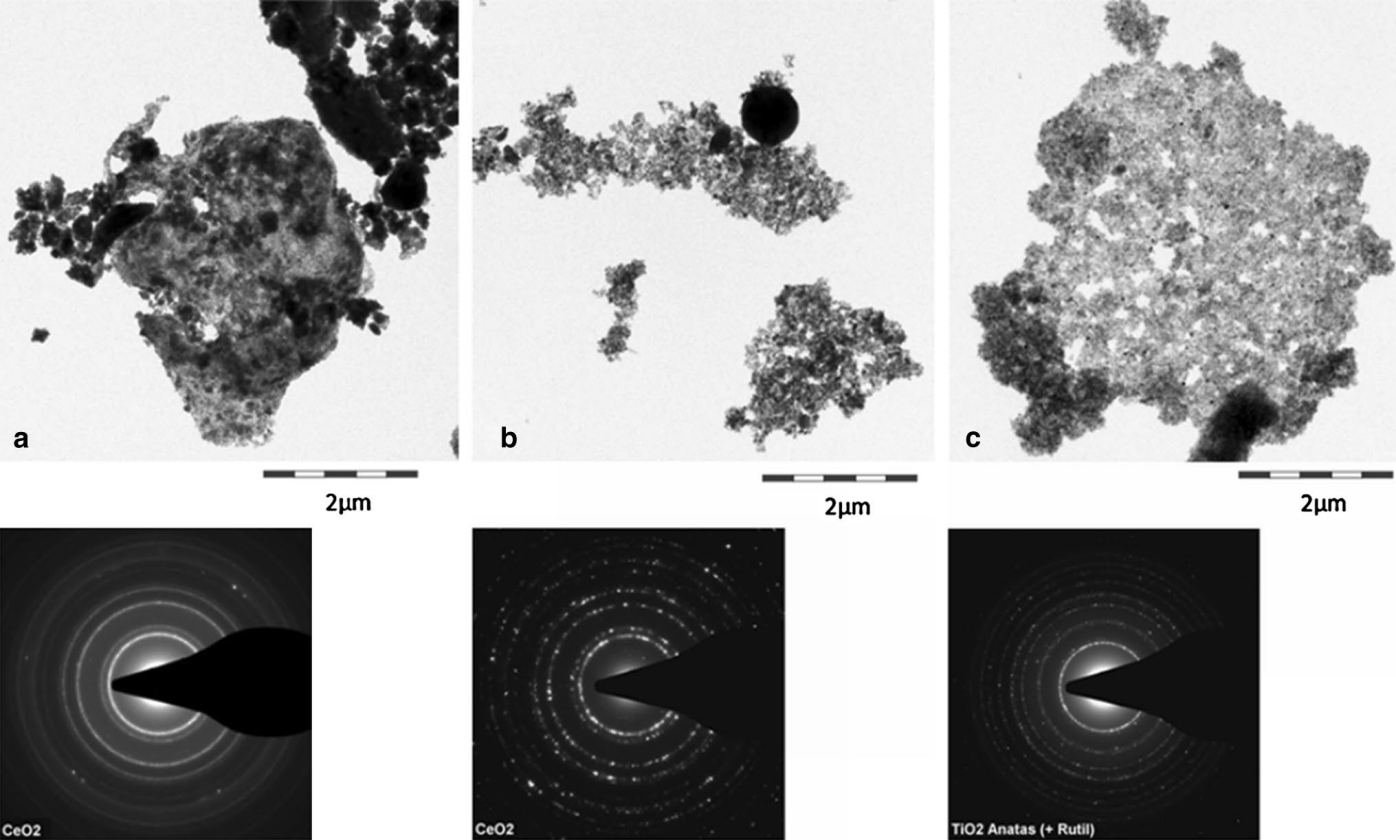
Persistence of Ceria NM-211 (**a**), NM-212 (**b**), and Titania NM-105 (**c**) as benchmark. Transmission electron microscopy (TEM) and selected area diffraction (SAD) after 28-day incubation in phagolysosomal simulant fluid (PSF) [see Table 1 for dissolution detected by ions in the supernatant (ICP-MS)]. The large almost spherical structures are no contaminations, but are confirmed as Ceria in the same crystalline phase

The solubility in physiological media was investigated by 28-day abiotic incubation in different fluids and detection of both the remaining particulate fraction (by SEM, centrifugation, LD, zeta-potential) and of the released metal ions (by ICP-MS). Both Ceria were insoluble except for marginal solubility in 0.1 N HCl (simulate oral ingestion), whereas a nanoscale precipitated Silica as positive control dissolved readily and Titania (NM-105) showed marginal solubility in phagolysosomal simulant fluid (PSF, simulate uptake and digestion in macrophages) (Wohlleben et al. 2013). In buffers with organic constituents (PSF, fasted state simulated intestinal fluid = FaSSIF), the surface charge reversed to negative zeta-potentials, which was identified earlier as indicator for the adsorption of constituents of the buffer for a range of the nano-Ceria (Wohlleben et al. 2013). Under all conditions, primary particles remained recognizable in TEM scans, but both Ceria NM-211 and NM-212 formed large structures after 28-day incubation in the acidic PSF medium that simulates the lysosome of macrophages (see Fig. 3). We found by selected area electron diffraction (SAD) that these aged structures retained the same cubic cerianite crystalline phase of the as-produced powder for both Ceria NM-211 and NM-212. Under identical conditions, also other nanomaterials, e.g., the Titania (NM-105), aged into similar structures, and we added the respective TEM and SAD for comparison (Wohlleben et al. 2013).

To summarize the comparative physical–chemical characterization, Ceria NM-211 and NM-212 had identical composition, agglomeration state, crystallinity, and insolubility. Compared to NM-212, the Ceria NM-211 had considerably smaller primary particles, larger specific surface, significantly fewer organic contaminations on the surface, and much reduced photocatalytic activity. Despite these differences, Ceria NM-211 and NM-212 shared the same tendency to recrystallize in PSF.

### Characterization of test atmosphere

The target concentrations were 0.5, 5, and 25 mg/m^3^ in the short-term studies (5 days and 4 weeks of exposure) with nano-Ceria NM-212. In the study with 5 days of exposure, 0.5 and 25 mg/m^3^ were selected for nano-Ceria NM-211. Analyzed concentrations and particle size distributions are summarized in Table 2. The target concentrations were met and maintained well throughout the studies. Particle size distribution demonstrated that Ceria particles were in the respirable range for rats.

**Table 2:** Measured concentrations and particle size distributions of Ceria

Study	Test substance	Targeted concentrations (mg/m^3^)	Measured concentrations mean ± SD (mg/m^3^)	MMAD (µm)/GSD mean	Particle count concentration measured by SMPS (particle/cm^3^)	Particle count median (nm)
5 days of exposure	Ceria NM-212	0.5	0.5 ± 0.2	1.4/2.3	2,583	283
		5	5.3 ± 0.9	1.2/2.1	137,096	206
		25	25.9 ± 6.0	1.0/2.5	537,548	171
	Ceria NM-211	0.5	0.48 ± 0.0	1.6/2.1	5,689	256
		25	5.2 ± 1.1	1.3/2.1	84,655	167
4 weeks of exposure	Ceria NM-212	0.5	25.6 ± 6.0	0.9/2.5	514,193	203
		5	0.45 ± 0.1	1.9/2.9	1,503	242
		25	25.8 ± 1.7	2.2/2.4	160,162	188

### Clinical examination

Five days and 4 weeks of inhalation exposure to Ceria NM-212 and NM-211 did not affect the body weight development of the animals (data not shown). The animals exposed to Ceria NM-211 and NM-212 showed no clinical signs or findings compared to the control animals (data not shown).

### Hematology, clinical chemistry, cytokines, and chemokines in serum

#### Five days of exposure

Absolute and relative neutrophil counts in blood were increased 3 days after the end of exposure to 25 mg/m^3^ Ceria NM-212 and NM-211, whereas relative lymphocyte counts were decreased (see Table S2). The changes were in a concentration-related manner. 24 days after the end of exposure, all parameters had returned to near control values. No other blood parameters were affected.

#### Four weeks of exposure

Hematological, clinical chemistry parameters, and acute-phase protein levels were not affected in rats exposed to Ceria NM-212 (see Table S2; the other data are not shown).

### Systemic genotoxicity (micronucleus test)

Micronuclei in the erythrocytes of peripheral blood cells were counted to assess chromosomal aberrations of hematopoietic cells in the bone marrow. Peripheral blood cells were examined in the short-term studies with 5 days and 4 weeks of exposure (3 and 2 days after the end of exposure, respectively). All examined parameters were in the range of control values (see Table S3).

### Bronchoalveolar lavage (BAL)

In the short-term studies with 5 days and 4 weeks of exposure, BALF analysis of five animals per test group was obtained 3 days or 1 day after the end of exposure and 24 or 35 days after the end of exposure, respectively (see Fig. 1). The resulting BALF parameters are presented in Table 3.

**Table 3:** Clinical pathology parameters in BALF of short-term studies with 5 days and 4 weeks of exposure

Target conc. (mg/m^3^)	5 days of exposure						4 weeks of exposure			
	Control	Ceria NM-212			Ceria NM-211		Control	Ceria NM-212		
	0	0.5	5	25	0.5	25	0	0.5	5	25
Measured conc. (mg/m^3^) + SD	0	0.48 ± 0.0	5.2 ± 1.1	25.6 ± 6.0	0.45 ± 0.1	25.8 ± 1.7	0	0.5 ± 0.2	5.3 ± 0.9	25.9 ± 6.0
BALF cell counts (cn/µL)										
Total cells										
Time point 1^a^	63.73 ± 11.03	69.76 ± 26.15	**98**.24** ± 16.38	**337**.35** ± 125.24	71.32 ± 22.69	**374**.98** ± 77.84	76.42 ± 23.97	75.10 ± 21.51	**133**.44* ± 48.40	**296**.90** ± 124.92
Time point 2^b^	53.09 ± 13.33	44.20 ± 2.45	43.98 ± 17.70	46.71 ± 8.16	47.01 ± 11.34	81.20 ± 38.87	75.29 ± 14.10	62.23 ± 11.59	97.44 ± 34.23	**220**.50** ± 105.27
Neutrophils (PMN)										
Time point 1^a^	0.82 ± 0.80	**3**.70** ± 2.53	**49**.19** ± 17.68	**272**.30** ± 106.62	**4**.41* ± 3.38	**297**.21** ± 59.23	0.85 ± 0.35	1.75 ± 1.13	**65**.66** ± 50.23	**222**.29** ± 99.25
Time point 2^b^	0.69 ± 0.38	1.34 ± 2.01	5.15 ± 9.91	**4**.63** ± 3.83	**2**.27** ± 0.56	**25**.57** ± 33.95	2.44 ± 1.01	2.52 ± 1.37	**41**.70** ± 22.0	**161**.69** ± 87.82
Lymphocytes										
Time point 1^a^	0.22 ± 0.32	0.17 ± 0.22	**1**.57* ± 0.97	**9**.20** ± 4.51	0.39 ± 0.34	**19**.14** ± 15.13	0.55 ± 0.50	0.63 ± 0.74	**5**.41** ± 3.21	**8**.93** ± 6.04
Time point 2^b^	0.32 ± 0.33	0.11 ± 0.10	0.43 ± 0.31	1.03 ± 0.95	0.51 ± 0.25	**2**.74** ± 2.17	1.65 ± 1.13	0.77 ± 0.49	**7**.43* ± 9.02	**10**.89** ± 3.48
Macrophages										
Time point 1^a^	62.65 ± 11.56	75.72 ± 15.10	46.83 ± 23.88	51.21 ± 24.76	66.47 ± 22.13	52.99 ± 42.89	74.94 ± 23.79	72.71 ± 21.16	60.25 ± 20.82	59.32 ± 26.28
Time point 2^b^	52.05 ± 13.00	42.32 ± 3.66	38.34 ± 11.92	40.93 ± 6.31	44.23 ± 11.53	52.61 ± 15.51	71.14 ± 13.31	58.78 ± 11.04	47.58 ± 16.48	45.17 ± 25.33
Monocytes										
Time point 1^a^	0.04 ± 0.08	0.00 ± 0.00	**0**.52* ± 0.37	**3**.**50** ± 1.86**	0.05 ± 0.11	**4**.40** ± 2.68	0.00 ± 0.00	0.00 ± 0.00	**1**.65* ± 1.90	**3**.95* ± 4.65
Time point 2^b^	0.00 ± 0.00	0.00 ± 0.00	0.03 ± 0.07	0.07 ± 0.11	0.00 ± 0.00	0.28 ± 0.34	0.06 ± 0.09	0.04 ± 0.08	**0**.68* ± 0.43	**2**.46* ± 3.03
Eosinophils										
Time point 1^a^	0.00 ± 0.00	0.00 ± 0.00	0.00 ± 0.00	0.39 ± 0.54	0.00 ± 0.00	0.00 ± 0.00	0.09 ± 0.12	0.00 ± 0.00	0.00 ± 0.00	0.34 ± 0.49
Time point 2^b^	0.03 ± 0.06	0.00 ± 0.00	0.03 ± 0.06	0.00 ± 0.00	0.00 ± 0.00	0.00 ± 0.00	0.00 ± 0.00	0.12 ± 0.11	0.05 ± 0.11	0.00 ± 0.00
Atypical cells										
Time point 1^a^	0.00 ± 0.00	0.00 ± 0.00	0.12 ± 0.17	0.76 ± 0.73	0.00 ± 0.00	**1**.25* ± 0.85	0.00 ± 0.00	0.00 ± 0.00	0.48 ± 0.72	**2**.07* ± 1.71
Time point 2^b^	0.00 ± 0.00	0.00 ± 0.00	0.00 ± 0.00	0.05 ± 0.07	0.00 ± 0.00	0.00 ± 0.00	0.00 ± 0.00	0.00 ± 0.00	0.00 ± 0.00	0.29 ± 0.40
Total protein/enzymes										
Total protein (mg/L)										
Time point 1^a^	39 ± 15	47 ± 16	75* ± 23	198** ± 52	44 ± 7	207** ± 54	60 ± 4	83** ± 5	94** ± 21	245** ± 77
Time point 2^b^	97 ± 122	59 ± 28	51 ± 16	60* ± 9	92 ± 91	82 ± 14	81 ± 23	60 ± 22	98 ± 49	175** ± 98
GGT (nkat/L)										
Time point 1^a^	20 ± 15	36 ± 20	106** ± 26	125** ± 15	37 ± 26	128** ± 16	37 ± 17	51 ± 11	111** ± 21	149** ± 31
Time point 2^b^	30 ± 7	28 ± 17	33 ± 14	61** ± 16	25 ± 10	87** ± 20	42 ± 12	44 ± 20	83** ± 30	123** ± 30
LDH (µkat/L)										
Time point 1^a^	0.46 ± 0.09	0.58 ± 0.17	**0**.89** ± 0.25	**2**.17** ± 0.21	0.67	**2**.60** ± 0.31	0.51 ± 0.18	0.55 ± 0.15	**1**.08** ± 0.37	**2**.28** ± 0.52
Time point 2^b^	0.36 ± 0.14	0.47 ± 0.25	0.42 ± 0.04	**0**.72** ± 0.20	0.58 ± 0.40	**1**.08** ± 0.32	0.58 ± 0.08	0.50 ± 0.20	**0**.84* ± 0.22	**1**.88** ± 1.20
ALP (µkat/L)										
Time point 1^a^	0.43 ± 0.10	0.53 ± 0.08	**1**.31** ± 0.53	**1**.56** ± 0.15	**0**.55* ± 0.09	**1**.56** ± 0.35	0.83 ± 0.16	0.84 ± 0.35	**1**.16** ± 0.10	**1**.53** ± 0.21
Time point 2^b^		1.0	**1**.5*	**1**.9**	0.51 ± 0.20	**0**.87** ± 0.12	0.70 ± 0.09	0.67 ± 0.08	**1**.05** ± 0.16	**1**.09** ± 0.25
NAG (nkat/L)										
Time point 1^a^	46 ± 9	47 ± 11	57 ± 12	74** ± 7	51 ± 19	94** ± 16	45 ± 5	55 ± 15	53* ± 8	86* ± 26
Time point 2^b^	41 ± 10	37 ± 12	39 ± 4	46 ± 7	46 ± 7	59* ± 10	47 ± 8	38 ± 6	47 ± 7	71 ± 35
Cell mediators (pg/mL)										
MCP-1										
Time point 1^a^	14.7 ± 0.6	19.1 ± 5.5	**101**.1** ± 31.3	**1**,**342**.9** ± 530.0	**17**.5* ± 1.6	**1**,**581**.0** ± 771.3	14.0 ± 0.0	19.6 ± 11.0	**559**.4** ± 444.4	**3**,**587**.2** ± 281.0
Time point 2^b^	48.2 ± 59.1	22.8 ± 12.5	26.7 ± 10.8	**144**.1* ± 82.8	30.6 ± 26.0	**329**.2** ± 290.5	17.3 ± 2.6	15.4 ± 3.1	**492**.5** ± 553.1	**1**,**854**.2** ± 1,184.0
CINC-1/IL-8										
Time point 1^a^	59.8 ± 17.7	**83**.4* ± 24.6	**348**.6** ± 185.4	**322**.4** ± 93.9	**88**.3* ± 21.1	**436**.0** ± 228.3	104.2 ± 26.7	103.8 ± 14.0	**506**.7** ± 195.9	**1**,**190**.9** ± 294.9
Time point 2^b^	88.3 ± 25.7	87.5 ± 24.7	94.3 ± 35.7	**155**.1** ± 13.5	99.7 ± 39.4	**254**.3** ± 70.4	158.8 ± 38.1	133.9 ± 45.3	**449**.4** ± 226.7	**831**.0** ± 497.1
M-CSF										
Time point 1^a^	41 ± 17	52 ± 23	34 ± 25	91** ± 28	61* ± 16	114** ± 48	26 ± 17	22 ± 12	27 ± 18	48 ± 29
Time point 2^b^	45 ± 19	48 ± 5	71* ± 13	49 ± 3	51 ± 12	60 ± 24	46 ± 26	55 ± 29	41 ± 14	53 ± 12
Osteopontin										
Time point 1^a^	172.28 ± 48.04	194.81 ± 131.24	301.40 ± 238.96	**849**.04** ± 386.44	134.32 ± 82.72	**1**,**116**.02** ± 653.35	391.44 ± 187.39	288.80 ± 110.90	**755**.44* ± 206.21	592.14 ± 336.47
Time point 2^b^	320.38 ± 177.64	191.47 ± 116.09	247.44 ± 189.46	495.92 ± 251.93	111.92 ± 80.28	398.72 ± 140.88	337.36 ± 282.91	284.40 ± 292.66	**1**,**003**.18* ± 434.20	838.48 ± 529.45

* Statistically significant, *p* < 0.05

** Statistically significant, *p* < 0.01; *n* = 5; *SD* standard deviation

^a^Time point 1: 3 days after the end of exposure (5 days of exposure) and 1 day after the end of exposure (4 weeks of exposure)

^b^Time point 2: 24 days after the end of exposure (5 days of exposure) and 35 days after the end of exposure (4 weeks of exposure)

#### Five days of exposure

In animals exposed to Ceria NM-212, the majority of BALF parameters were increased at aerosol concentrations of 5 mg/m^3^. At 0.5 mg/m^3^, the neutrophil counts and cytokine-induced neutrophil chemoattractant-1 (CINC-1) were both statistically increased and were slightly above the historical control range (see Table S4). With Ceria NM-211, but not NM-212, monocyte chemoattractant protein-1 (MCP-1) and macrophage colony-stimulating factor (M-CSF) were increased at aerosol concentrations of 0.5 mg/m^3^ and above. 24 days after the end of exposure, a full recovery was observed at aerosol concentrations of 0.5 mg/m^3^ and a partial recovery at aerosol concentrations of 5 and 25 mg/m^3^. The recovery of animals exposed to 25 mg/m^3^ Ceria NM-211 seems to be slower than those exposed to NM-212.

#### 4 weeks of exposure

Four weeks of inhalation exposure to 5 and 25 mg/m^3^ Ceria NM-212 resulted in an increase in total cells in BALF due to increases in polymorph nuclear neutrophils, lymphocytes, and monocytes in BALF (see Table 3). Consistent with these findings, several other parameters including the examined cell mediators were increased. 35 days after the end of exposure, some of the BALF parameters returned to control levels, whereas several of them were still significantly increased at 5 and 25 mg/m^3^ (e.g., total cells, lymphocytes, neutrophils; GGT, LDH, ALP; MCP-1, CINC-1).

**Fig. 4:**
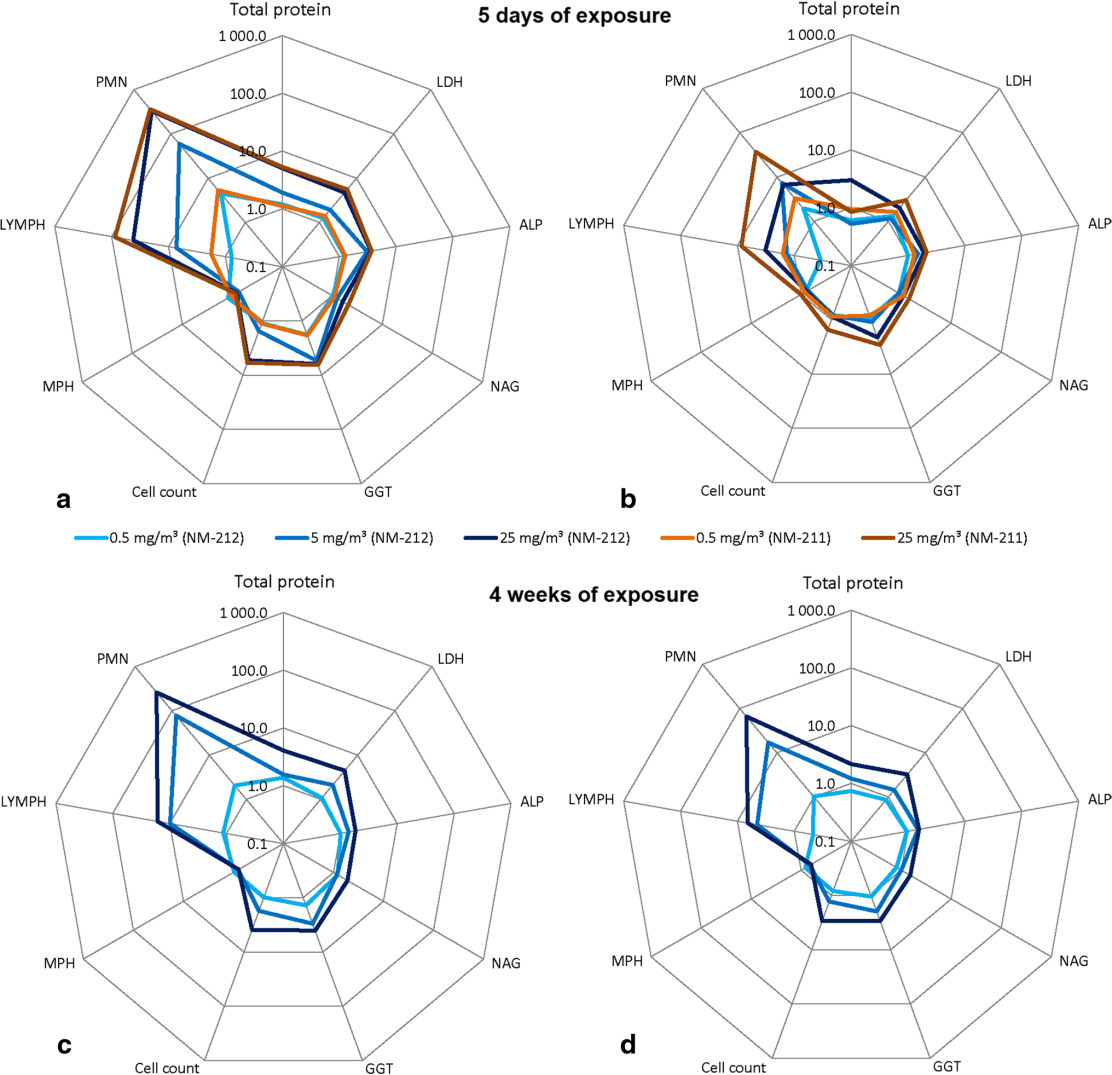
Comparison of changes in BALF parameters after 5 days (**a**, **b**) and 4 weeks (**c**, **d**) of exposure to Ceria NM-211 and NM-212: short-term study with 5 days of exposure: **a** 3 days after the end of exposure, **b** 24 days after the end of exposure; short-term study with 4 weeks of exposure, **c** 1 day after the end of exposure, **d** 35 days after the end of exposure. Changes are shown as *x*-fold differences compared to controls using a logarithmic scaling

Five days of exposure caused slightly higher neutrophil and lymphocyte counts at aerosol concentrations of 25 mg/m^3^ Ceria NM-212 compared to 4 weeks of exposure (see Fig. 4). CINC-1 was already increased at 0.5 mg/m^3^ after 5 days but not after 4 weeks of exposure. The regression of the BALF parameters was faster after 5 days than after 4 weeks of inhalation exposure. Cell mediators, especially MCP-1, were higher elevated at concentrations of 5 and 25 mg/m^3^ after 4 weeks than after 5 days of exposure.

### Pathology

#### Organ weights and macroscopic findings

*Five days of exposure* After 5 days of exposure, no increase in lung weights was observed after exposure to Ceria NM-212 (see Table S5). An aerosol concentration of 25 mg/m^3^ Ceria NM-211, however, resulted in significant increases in absolute and relative lung weights (+20 and 24 %, respectively, *p* < 0.01). The increase in lung weight was no longer present 21 days after the end of exposure. After 5 days of exposure, the macroscopic finding enlarged mediastinal lymph nodes were found in individual animals of all test groups (see Table S5). 21 days after the end of the exposure, enlarged mediastinal lymph nodes were observed in almost all animals exposed to Ceria NM-211 or NM-212. The tracheobronchial lymph nodes and all other examined organs did not show any macroscopic findings.

*Four weeks of exposure* Absolute and relative lung weights were significantly increased at aerosol concentrations of 25 mg/m^3^ Ceria NM-212 (+30 and 29 %, respectively) 2 days after the end of the exposure and were still significantly elevated (+16 and 20 %) 34 days after the end of the exposure (see SI, S5). 2 days after the end of the exposure, absolute and relative lung weights of animals exposed to 5 mg/m^3^ were increased significantly by +13 and 10 %, respectively. They returned to control levels within the following 34 days. No effects on organ weights were observed after inhalation of 0.5 mg/m^3^. 2 days after the end of exposure, mediastinal lymph nodes of two animals (out of ten) were enlarged at aerosol concentrations of 25 mg/m^3^ Ceria NM-212. 34 days after the end of exposure, the incidence of animals with enlarged, yellow white-colored mediastinal lymph nodes increased considerably from two to eight (out of ten) per group at aerosol concentrations of 25 mg/m^3^ Ceria NM-212, respectively. Mediastinal lymph nodes of animals exposed to 5 mg/m^3^ were firstly enlarged 34 days after the end of exposure.

All other extrapulmonary organs including the tracheobronchial lymph nodes revealed no macroscopical findings after inhalation exposure to Ceria.

### Histopathology

**Fig. 5:**
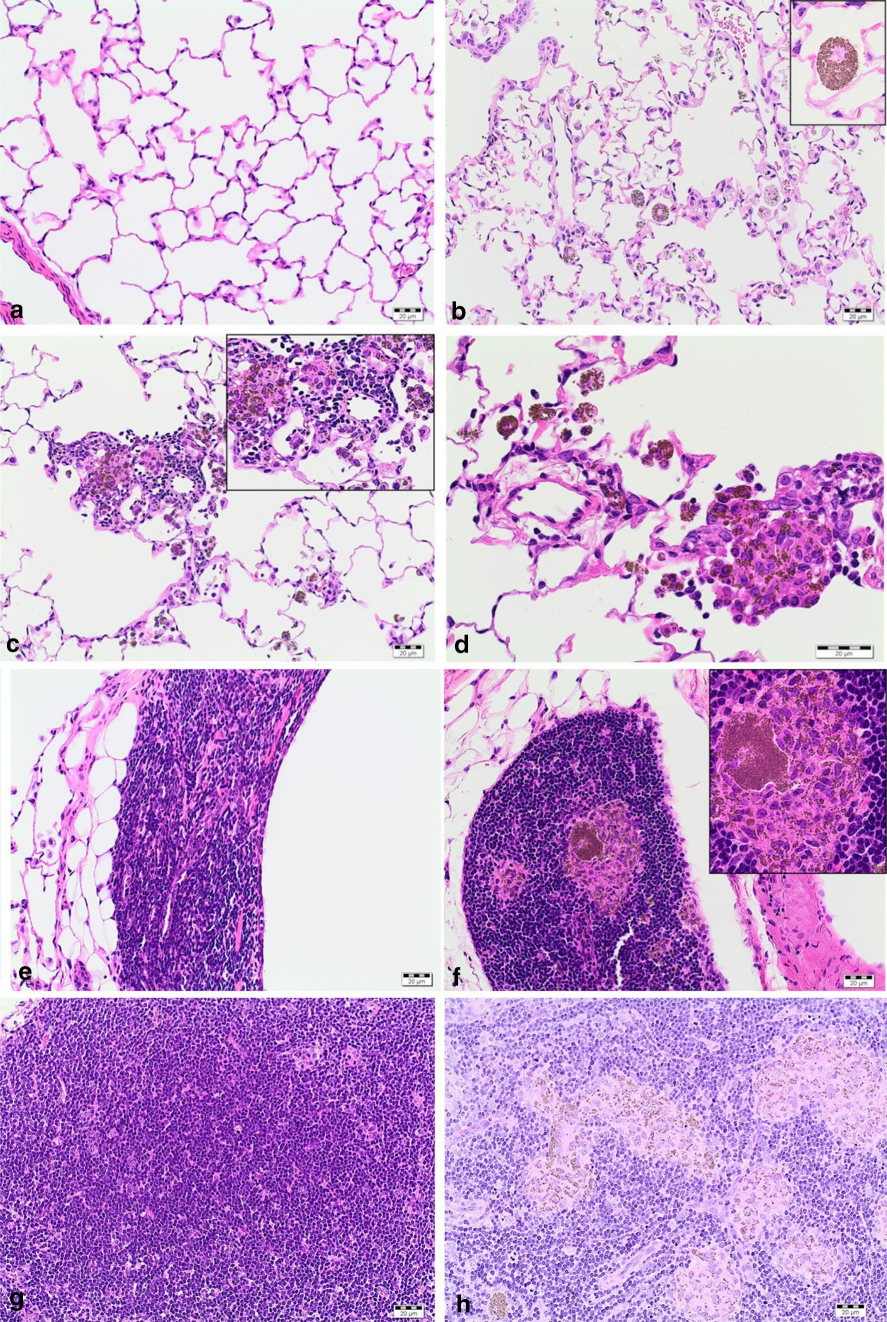
**a** Microscopic appearance of lungs (**a**–**d**) in the short-term study with 4 weeks of exposure. **a** Control, lung; **b** 2 days after the end of exposure to 25 mg/m^3^ Ceria NM-212: lung, alveolar histiocytosis with particles; **c**, **d** 34 days after the end of exposure to 25 mg/m^3^ Ceria NM-212: lung, granulomatous inflammation. **b** Microscopic appearance of lungs (**e**, **f**) in the short-term study with 4 weeks of exposure; **e** control, BALT; **f** 34 days after the end of exposure to 25 mg/m^3^ Ceria NM-212: BALT macrophage aggregates with particles. **c** Microscopic appearance of lung-associated lymph nodes (**g**, **h**) in the short-term study with 4 weeks of exposure; **g** control, lymph node; **h** 34 days after the end of exposure to 25 mg/m^3^ Ceria NM-212: tracheobronchial lymph node, macrophage aggregation with particles

Overall, histopathological evaluations were consistent in the short-term studies with 5 days and 4 weeks of exposure. The findings assessed in the short-term study with 5 days showed lower severities (see Table 4; Fig. 5a–c).

**Table 4:** Histopathology of short-term studies with 5 days and 4 weeks of exposure

Target conc. (mg/m^3^)	5 days of exposure to Ceria NM-212						5 days of exposure to Ceria NM-211				4 weeks of exposure to Ceria NM-212					
	Time point 1^a^			Time point 2^b^			Time point 1^a^		Time point 2^b^		Time point 1^a^			Time point 2^b^		
	0.5	5	25	0.5	5	25	0.5	25	0.5	25	0.5	5	25	0.5	5	25
Histopathology																
**Lungs**																
Alveolar histiocytosis with particles	–	–	**5**	–	**3**	**5**	–	**5**	–	**4**	**10**	**10**	**10**	–	**10**	**10**
Grade 1			1		3	5		3		4	10	4			3	
Grade 2			4					2				6	5		7	
Grade 3													5			10
Eosinophilic granular material/particles	–	–	**5**	–	–	–	–	**5**	–	–	–	**10**	**10**	–	**2**	**10**
Grade 1			2					5				10			2	5
Grade 2			3										6			4
Grade 3													4			1
BALT: macrophage aggregates particles	–	–	–	–	–	–	–	–	–	**1**	–	–	**5**	–	**1**	**8**
Grade 1										1			5			3
Grade 2															1	2
Grade 3																3
BALT: occurrence of particles	–	**2**	**5**	–	**3**	**5**	–	**5**	**2**	**4**	–	**8**	**5**	**3**	**8**	**2**
Inflammation, granulomatous	–	–	–	–	–	–	–	–	–	–	–	–	–	–	**1**	**5**
Grade 1															1	
Grade 2																5
Particles within histiocytes	**5**	**5**	–	**5**	**2**	–	**5**	–	**5**	–	**–**	–	–	**10**	–	–
**Mediastinal lymph nodes**																
Macrophage aggregates particle.	–	–	–	–	–	**4**	–	–	–	**2**	–	–	**4**	–	**9**	**9**
Grade 1						2				1			2			
Grade 2						1									2	
Grade 3										1			2		6	2
Grade 4						1									1	7
Occurrence of particles	–	–	**2**	–	**1**	**1**	–	**3**	–	**2**	–	**6**	**6**	**1**	**–**	**1**
Hyperplasia, lympho-reticulocellular	**1**	–	**3**	–	–	**3**	**3**	**3**	–	**1**	**1**	**1**	**4**	–	**2**	**7**
Grade 1	1		3			2	3	1					1			
Grade 2						1		1		1	1	1	3		2	6
Grade 3								1								1
**Tracheobronchial lymph nodes**																
Macrophage aggregates particle.	–	–	–	–	–	**4**	–	–	–	**3**	–	**1**	**8**	–	**10**	**10**
Grade 1										1		1	3		1	1
Grade 2						2				1			5		1	1
Grade 3						2									5	2
Grade 4										1					3	6
Occurrence of particles	–	–	**5**	–	**3**	**1**	–	**4**	–	**2**	–	**7**	**2**	**1**	–	–
Hyperplasia, lympho-reticulocellular	–	–	**4**	**1**	**1**	**2**	–	**1**	**1**	**1**	–	**1**	**7**	–	**1**	**6**
Grade 1			4					1				1	2		1	1
Grade 2				1	1	2			1	1			3			4
Grade 3													2			1

Number of examined organs per test group: *n* = 10 (4 weeks of exposure); *n* = 5 (5 days of exposure)

Grade 1: minimal, grade 2: slight, grade 3: moderate, grade 4: severe, grade 5: extreme; whenever a grading was not used, the microscopic finding was indicated to be present

^a^Time point 1: after the end of exposure (5 days of exposure) and 2 days after the end of exposure (4 weeks of exposure)

^b^Time point 2: 21 days after the end of exposure (5 days of exposure) and 34 days after the end of exposure (4 weeks of exposure)

### Lungs

#### Five days of exposure

After 5 days of exposure, single or accumulated macrophages were located in the lumen of the alveoli and a few macrophages occurred also in the alveolar wall and ducts. They were distributed multifocally in all lobes over the whole lung. Most of them were loaded with amber-like colored particles of different sizes (below 1 µm diameter). These particles were considered to represent agglomerated or aggregated Ceria particles (“alveolar histiocytosis with particles”). Alveolar histiocytosis and free eosinophilic granular material with particles, interpreted as remnants of destroyed macrophages, were found in the lung of almost all animals exposed to 25 mg/m^3^ Ceria NM-211 and NM-212. At aerosol concentrations of 0.5 mg/m^3^ Ceria NM-211, 0.5 and 5 mg/m^3^ Ceria NM-212, amber-colored particles were noted within single histiocyte in the alveoli. In the bronchus-associated lymphoid tissue (BALT), particles were detected free or in single macrophages, mostly at 5 mg/m^3^ Ceria and above. Findings regressed but were still present 21 days after the end of exposure. The finding “eosinophilic granular material with particles” was no longer visible after the post-exposure period. Particles were detected free or in single macrophages in BALT, even at the lowest concentration of 0.5 mg/m^3^ Ceria NM-211. Only one animal exposed to 25 mg/m^3^ Ceria NM-211 showed macrophage aggregates in BALT 21 days after the end of exposure.

#### Four weeks of exposure

Two days after the end of the exposure, a concentration-related increase in alveolar macrophages was observed in the lungs in all Ceria NM-212-exposed animals (Table 4; Fig. 5a). Eosinophilic granular material and small particles were distributed in the alveoli of animals exposed to 5 and 25 mg/m^3^ Ceria NM-212. The occurrence of alveolar histiocytosis and of eosinophilic granular material was correlated with increased lung weights in animals exposed to 5 and 25 mg/m^3^ Ceria NM-212. In BALT, single, small macrophage aggregates with particles occurred in animals exposed to 25 mg/m^3^ (see Fig. 5b). In addition, single or a few amber-like colored particles occurred extracellularly in BALT without any macrophage activation at aerosol concentrations of 5 and 25 mg/m^3^. 34 days after the end of exposure, alveolar histiocytosis and eosinophilic granular material with particles were still observed at concentrations of 5 and 25 mg/m^3^ Ceria NM-212. At 0.5 mg/m^3^, in contrast, amber-like colored particles could only be noted within single histiocyte. In one animal of 5 mg/m^3^ and in five out of ten animals exposed to 25 mg/m^3^ Ceria NM-212, a multifocal granulomatous inflammation appeared first. In BALT, single or few amber-colored particles at 0.5, 5, and 25 mg/m^3^ Ceria NM-212 as well as an increasing number of animals with macrophage aggregates with particles at 5 and 25 mg/m^3^ were still observed (see Fig. 5b). All compound-related findings after exposure of 5 and 25 mg/m^3^ Ceria NM-212 were correlated with increased lung weights in these test groups.

### Lung-associated lymph nodes (mediastinal and tracheobronchial lymph nodes)

#### Five days of exposure

After 5 days of exposure to 25 mg/m^3^ Ceria NM-211 and NM-212, comparable to the finding of particles in BALT of the lung, amber-like colored particles were seen partly within macrophages or extracellularly in the lymphoid tissue, without any macrophage activation or aggregation. 21 days after the end of exposure to 25 mg/m^3^ Ceria NM-211, findings in both lymph nodes progressed. In the mediastinal and the tracheobronchial lymph nodes, multifocal macrophage aggregates with amber-like colored particles were noted in animals exposed to Ceria NM-211 and NM-212 (see Table 4). In both lymph nodes, lympho-reticulocellular hyperplasia was observed after 5 days of exposure and 21 days after the end of exposure to Ceria NM-211 and NM-212.

#### Four weeks of exposure

Two days after the end of exposure to 5 and 25 mg/m^3^ Ceria NM-212, multifocal macrophage aggregates with particles were observed in the mediastinal as well as in the tracheobronchial lymph nodes (see Fig. 5c). A lympho-reticulocellular hyperplasia was present in both lymph nodes, mostly seen in animals in the group of 25 mg/m^3^. The hyperplasia of the mediastinal lymph nodes was correlated with their corresponding macroscopic enlargement after exposure to 25 mg/m^3^ Ceria NM-212. 34 days after the end of exposure, the number of animals with macrophage aggregates (incidence and grading) and with hyperplasia in both lymph nodes was higher compared to the animals examined 2 days after the end of the exposure. Nearly all other findings were still present 34 days after the end of the exposure.

### Upper respiratory tract

#### Five days of exposure

After 5 days of exposure to 25 mg/m^3^ Ceria NM-211 and NM-212, extracellular, amber-like colored particles with diameters up to 1.5 µm were found in the lamina propria mucosae of the dorsal area of the larynx (level III). At aerosol concentrations of 25 mg/m^3^ Ceria NM-211 and NM-212, resembling amber-like colored particles were detected within the subepithelial tissue in the carina of the trachea. 21 days after the end of exposure, findings in the larynx were only observed for animals exposed to Ceria NM-212, whereas particles in the carina (trachea) were still present in animals exposed to Ceria NM-211 and NM-212.

#### Four weeks of exposure

Two days after the end of the exposure to 5 and 25 mg/m^3^ Ceria NM-212, amber-like colored particles occurred similarly in the dorsal area of the larynx (level III). Animals exposed to 25 mg/m^3^ showed particles in the carina of the trachea. At aerosol concentrations of 25 mg/m^3^ Ceria NM-212, single amber-like colored particles were firstly found in the nasal-associated lymphoid tissue (NALT), inside single macrophages or extracellularly. These findings were still present 34 days after the end of exposure.

### Extrapulmonary organs

#### Five days of exposure

Extrapulmonary organs of animals exposed for 5 days to Ceria NM-211 and NM-212 were not examined.

#### Four weeks of exposure

Histological examination of extrapulmonary organs, e.g., liver, spleen, and kidneys, did not show any substance-related morphological changes in animals exposed to 0.5, 5, and 25 mg/m^3^ Ceria NM-212 in the short-term study with 4 weeks of exposure (data not shown).

### Organ burden analysis

**Fig. 6:**
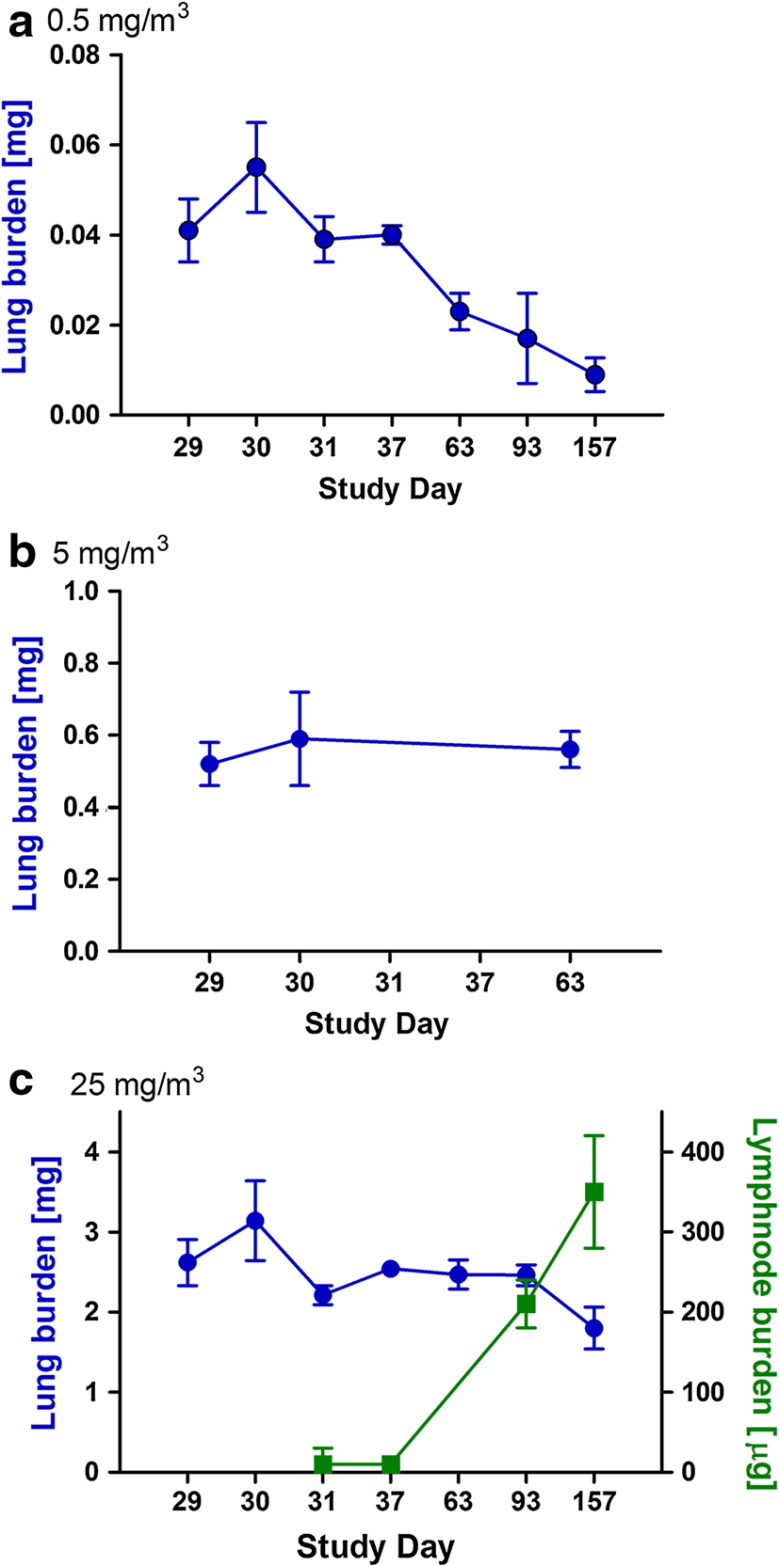
Biokinetics of lung (**a**–**c**) and lymph node burdens (**c**) in the short-term study with 4 weeks of exposure. Lung burden after exposure to 0.5 mg/m^3^ (**a**), 5 mg/m^3^ (**b**), and 25 mg/m^3^ (**c**) Ceria NM-212 and burden of lung-associated lymph nodes after exposure to 25 mg/m^3^ Ceria NM-212 (**c**, *green*) after 4 weeks of exposure and during 129 days post-exposure

In the short-term studies with 5 days and 4 weeks of exposure, Ceria burden of lung, lymph nodes, and partly liver is summarized in Tables S6–S8. The time course of lung and lymph node burdens is presented in Fig. 6.

#### Five days of exposure

Exposure to 0.5 mg/m^3^ Ceria NM-212 resulted in a lung burden of 0.011 mg/lung, directly after 5 days of exposure and decreased to 0.006 mg/lung 21 days after the end of the exposure, whereas exposure to 5 and 25 mg/m^3^ yielded higher lung burdens (0.1 and 0.53 mg/lungs, respectively) with only little decrease (0.088 and 0.4 mg/lung, respectively) within 21 days after the end of the exposure.

Lung burdens of Ceria NM-211 were around twofold lower compared to those of Ceria NM-212 (see Table S6). Cerium content in the lung-associated lymph nodes at aerosol concentrations of 25 mg/m^3^ increased from 1.7 to 5 µg for Ceria NM-212 and from 1.4 µg to 3 µg for Ceria NM-211 in the short-term study with 5 days of exposure (see Table S7).

#### Four weeks of exposure

The time course of the lung burdens is presented in Fig. 6 (for detailed values, see Table S6). Inhalation exposure of 0.5, 5, or 25 mg/m^3^ Ceria NM-212 resulted in mean lung burdens of 0.04, 0.52, or 2.62 mg 1 day after the end of exposure. 2 days after the end of exposure, higher lung burdens of the left lungs were measured and these data were disregarded for half-time calculations. Applying the following equation, retention half-times of Ceria in the lungs were calculated as

$$N(t) = N_{0} \times {\text{e}}^{ - \lambda t}$$

where *N*(*t*) was the lung burden at time point *t*, N_0_ was the lung burden shortly after last exposure, *t* as days after last exposure.

At aerosol concentrations of 0.5 mg/m^3^ Ceria NM-212, a retention half-time of 40 days was determined. Higher aerosol concentrations of 25 mg/m^3^ Ceria NM-212 resulted, however, in a much longer half-time above 200 days.

The Cerium burden in the lung-associated lymph nodes (tracheobronchial and mediastinal lymph nodes) was 10 µg 3 days after the end of the exposure to 25 mg/m^3^ Ceria NM-212 and increased to 350 µg 129 days after the end of exposure (see Table S7).

After the exposure to 25 mg/m^3^ Ceria NM-212, Cerium was also detected in the liver (1.56 and 1.93 µg) 3 and 65 days after the end of the exposure, respectively (see Table S8).

## Discussion

Objectives of this body of work were to determine lung deposition and clearance kinetics as well as the inhalation toxicity of two nano-Ceria (NM-211 and NM-212) in short-term studies with 5 days and 4 weeks of inhalation exposure. The results should serve as basis for selecting appropriate exposure concentrations for a subsequent long-term inhalation study. The following sections focus on a discussion of (1) deposition and clearance, (2) local and systemic effects, (3) comparison of the two Ceria NM-211 and NM-212, and (4) comparison of 5 days and 4 weeks of exposure.

### Deposition and clearance

Four weeks of exposure to 0.5 mg/m^3^ Ceria NM-212 resulted in a lung burden of 41 µg/lung; the post-exposure retention half-time was 40 days. This is in the range of physiological retention half-times of poorly soluble particles between 60 and 70 days (Oberdorster 2002). A higher aerosol concentration of 25 mg/m^3^ elicited a lung burden of 2,620 µg/lung resulting in a retarded retention half-time above 200 days. At the mid concentration of 5 mg/m^3^, the lung burdens at three time points indicated a retarded retention half-time as the lung burden (500 µg/lung) stayed at a constant level during 4 weeks of post-exposure. A prolonged lung clearance of the same Ceria NM-212 was also observed in a rat instillation study with neutron-activated ^141^Ceria NM-212 at an instilled dose of 1 mg/kg body weight, corresponding to 200–300 µg/lung (Molina et al. 2014). During 28 days post-exposure, only 12 % of the ^141^Cerium in the lung has been cleared.

Pulmonary inflammation was only observed at concentrations of 5 and 25 mg/m^3^, which also caused significant retardation of pulmonary clearance. Concerning the threshold for overload conditions, mass lung burden of 41 µg, achieved at 0.5 mg/m^3^, was well below the overload threshold proposed by Morrow, while the lung burden of 2,620 µg, achieved at 25 mg/m^3^, was above it (Morrow 1988). At 25 mg/m^3^; a strong pulmonary inflammation was apparent. The mid concentration of 5 mg/m^3^ Ceria NM-212 elicited pulmonary inflammation at a constant lung burden of around 520 µg, which is slightly below or at the border of the overload threshold.

**Fig. 7:**
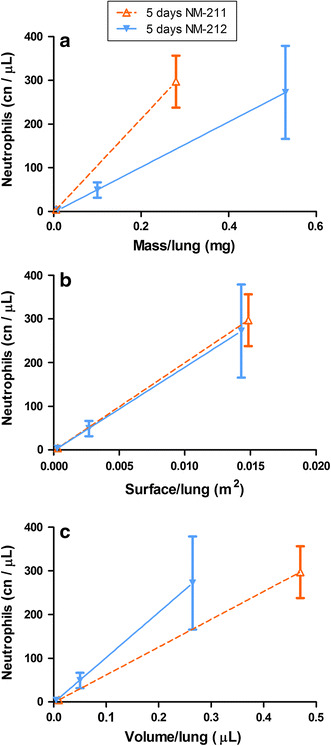
Dose–response curves using different dose metrics after 5 days of exposure to 0.5 and 25 mg/m^3^ Ceria NM-211 and 0.5, 5, and 25 mg/m^3^ Ceria NM-212; **a** mass, **b** surface, **c** volume

Morrow assumed overload conditions when particle volume exceeds 60 µm^3^/alveolar macrophage (Morrow 1988). Oberdoerster et al. (1994a) suggested retained surface area as appropriate metrics for correlating overload with retarded clearance, particularly if nanoparticles are involved. Tran et al. (2000) proposed overload threshold values of 0.02–0.03 m^2^/g lung. In the current study, 4 weeks of exposure to an aerosol concentration of 25 mg/m^3^ caused a particle surface burden of 0.07 m^2^/lung and 5 mg/m^3^ resulted in 0.014 m^2^ surface burden per lung (see Fig. 7; Table S9). This is slightly below the surface burden threshold proposed by Tran et al. and caused already inflammation and retarded clearance in the lung. Therefore, it can be assumed that a threshold, however, is highly material-dependent and may not be based on one or two particle types alone. The thresholds published previously may need adaptations for the specific type of nanomaterial in order to address its toxicological properties.

Lung burdens after 5 days of exposure were about one-fourth of those after 4 weeks of exposure reflecting a linear kinetics. This is the consequence of a linear deposition and a slow clearance. In the long term, however, the kinetics will not be linear due to clearance.

In the lung-associated lymph nodes, 350 µg Ceria was found 129 days after the exposure to 25 mg/m^3^ for 4 weeks. The lung burden decreases from 2,620 to 1,800 µg in the same time period. The lymphatic clearance of Ceria was around 13 % of the initial retained burden after the end of the exposure. Lymphatic clearances of 1–5 % were previously reported for microscale particles but were also shown to be higher for nanoscale particles (Kreyling and Scheuch 2000; Kreyling et al. 1986; Oberdorster et al. 1994b). Most of the Ceria nanoparticles were presumably cleared by mucociliary clearance and subsequent fecal excretion, which was not evaluated in this work.

A very low content of Ceria was detected in the liver in the presented inhalation study with 4 weeks of exposure to 25 mg/m^3^ Ceria NM-212. Consistent with our findings, accumulation of Ceria NM-212 in extrapulmonary tissue and associated toxicity appeared to be low as reported in a rat study using intravenous injection of neutron-activated ^141^Cer NM-212 (Molina et al. 2014). Moreover, dissolution of Ceria within alveolar macrophages or other extracellular fluids, and subsequent absorption/bioavailability of ionic Cerium into the blood and transport to extrapulmonary organs, may occur (Oberdörster et al. 2005). The exploration in simulant fluids indicated structural changes mediated by marginal solubility in (acidic) PSF, but not in (neutral) PBS (see Table 1). Hence, there is only a minor, if any, contribution of ionic Cerium.

### Local effects

Local pulmonary effects after inhalation of nano-Ceria were consistent after 5 days and 4 weeks of exposure. The reported pulmonary inflammation was assessed by the changes in BALF parameters (e.g., neutrophils) and histopathological findings (alveolar histiocytosis and granulomatous inflammation).

In BALF, proinflammatory cytokines are also indicators of an inflammatory process in the lung (Henderson 1984). Increased activities of the enzymes ALP and NAG indicated the proliferation of pneumocyte type II cells and activation of macrophages, respectively (Henderson 2005; Capelli 1997). Consistently, the chemokine MCP-1 released by macrophages was concentration-related increased indicating a recruitment of blood monocytes into the alveolar compartment. The pro-inflammatory and neutrophil-attractant chemokine CINC-1 were increased in parallel but did not exceed MCP-1. Both parameters were assessed as predictive markers for early pulmonary inflammation and macrophage recruitment into the lungs in previous inhalation studies testing 14 different nanomaterials (Landsiedel et al. 2014). However, MCP-1 was the only cell mediator, which could be correlated with the progression of alveolar histiocytosis in histopathology. Consistently to ascending gradings and incidences in histopathology after 4 weeks exposure, MCP-1 levels were higher increased after 4 weeks than 5 days of exposure. The other cell mediators were not able to predict or indicate any of the observed morphological changes. The cytokine M-CSF is a marker for the differentiation of monocytes into histiocytes. Produced by macrophages, it may be responsible for increases in macrophage numbers (Landsiedel et al. 2014), but was not increased overall. Osteopontin is known to be involved in pulmonary granuloma development in rodents (Chiba et al. 2000), but was not increased in our studies, although a granulomatous inflammation was observed.

In BALF, macrophages counts were not elevated significantly. However, increase in alveolar macrophage number, different stages of (recruited, activated, highly particle loaded, destroyed) macrophages, and granulomatous inflammation was apparent in histopathology. This discrepancy between macrophage numbers in BALF and histopathology during an inflammatory state in the lungs was already recognized in different other inhalation studies (Porter et al. 2002, 2007). Most probably, the lung macrophages were not detached and washed out using the lavage technique with only two washes in our studies (Henderson 2005). Other studies with Ceria reported higher macrophage counts in BALF using the same or a higher number of washes (Gosens et al. 2013; Ma et al. 2011). The BAL method used in the here-presented studies is highly standardized and yields highly reproducible results compared to more rigorous lavage techniques but is obviously not capable of detaching more sessile macrophages of the lung. Hence, neutrophil counts in BALF were used as sensitive parameter to indicate an inflammatory response (Henderson 2005; Henderson et al. 1979a, b).

In pathology, lung weights were increased in the studies with 5 days (25 mg/m^3^ Ceria NM-211) and 4 weeks (5 and 25 mg/m^3^ Ceria NM-212) of exposure. By light microscopy, Ceria particles were primarily seen extracellularly and intra-alveolar or engulfed by alveolar macrophages. Different to observations after inhalation to other nanoparticles such as Titania, Ceria was not detected within alveolar epithelial cells in the current study (Warheit and Hartsky 1993). This supports the hypothesis that macrophage activation and immobilization together with cytokine and fibrogenic effects may be the mode of action for microscale Ceria inhalation toxicity [Environmental Protection Agency (EPA) 2009]. Furthermore, apoptosis of alveolar macrophages, their stimulation (pro-inflammatory cytokine and ROS release), and switch from the inflammatory M1 to a fibrotic M2 type was shown by the interaction of nano-Ceria with these cells after an intratracheal instillation study with rats (Ma et al. 2011).

At the lowest concentration of 0.5 mg/m^3^ in the present studies, the histopathological findings alveolar histiocytosis and particles, either free or within macrophages, reflect an expected physiological response (Brain et al. 1976). However, after 4 weeks of exposure, the alveolar histiocytosis had progressed to a multifocal granulomatous inflammation at 5 mg/m^3^ and at 25 mg/m^3^ within 4 weeks after the end of exposure. The combination of moderate alveolar histiocytosis with particles and the presence of eosinophilic material, potentially precursors of granulomatous inflammation, are thus considered to be adverse. Granulomatous inflammation, multifocal microgranulomas, or granulomas due to inhalation of nano-Ceria have previously been reported in a number of animal studies (Srinivas et al. 2011; Cho et al. 2010; Snipes 1989; Aalapati et al. 2014). Granulomatous inflammation represents one type of chronic inflammation which is characterized by inflammatory lesions with large numbers of macrophages forming interlacing palisades, lymphocytes, and plasma cells (Renne et al. 2009). Other cells of the mononuclear phagocyte system (epithelioid and multinucleated cells) also contribute to maintain the inflammatory process. However, in the present studies, later stages of granuloma formation, transformation of macrophages to epithelioid, or giant cells were not observed. However, granulomatous inflammation can lead to tissue damage and fibrosis at later stages as observed by Ma et al. 4 weeks after a single intratracheal instillation of 7 mg/kg nano-Ceria in rats (Ma et al. 2014).

Concerning the two different methods (BAL and histopathology) in the study with 4 weeks of exposure, BALF parameters showed a regression during the post-exposure period. Histopathological findings, in contrast, progressed after the end of the exposure at concentrations of 5 mg/m^3^ Ceria and above. A regression of the BALF parameter (e.g., neutrophils) and a certain progression of histopathological findings during the post-exposure were already recognized in previous works with Ceria (Gosens et al. 2013; Srinivas et al. 2011). It was concluded that during a sustained inflammation in the lungs, parameters in BALF do not represent the current inflammatory state and damage in the lungs.

Lymphatic clearance of inhaled Ceria via the lymphatic vessels from the pulmonary region to the lung-associated lymph nodes was demonstrated by histological evaluations and confirmed by measured Cerium lymph node burdens. Accumulation of free particles or particles within macrophage aggregates was detected in the BALT or mediastinal and tracheobronchial lymph nodes. Increasing Cerium clearance into the lung-associated lymph nodes was correlated with an increase in grading and severity of macrophage aggregates during the post-exposure period (see pathological score, Table 4). Moderate macrophage aggregates with particles in the lung-associated lymph nodes, combined with lympho-reticulocellular hyperplasia, were considered to be adverse. It is more likely that the particles reached the associated lymph nodes by phagocytizing macrophages than by entering the interstitium.

Aerosol concentrations of 5 and 25 mg/m^3^ caused inflammatory effects in the lung, which can be considered adverse. At 0.5 mg/m^3^, slight effects were still detected by BALF after 5 days of exposure but not by histopathology or after 4 weeks of exposure. BALF parameters proved to be more sensitive to short-term effects but less predictive for the progression of these effects.

The observations of the current study resemble the findings of previously published inhalation (and instillation) studies with different Ceria (Gosens et al. 2013; Ma et al. 2011; Demokritou et al. 2012; Aalapati et al. 2014). In an 28-day inhalation study with the Ceria NM-212, Gosens et al. (2013) observed pulmonary effects at an aerosol concentration equivalent to 2.5 mg/m^3^. These effects were less pronounced compared to the current study. The dose-equivalent concentration of 2.5 mg/m^3^ was achieved by the exposure of the animals to 50 mg/m^3^ for different daily exposure durations, whereas the studies reported here exposed animals to actual 0.5, 5, and 25 mg/m^3^ for 6 h/day. The difference in daily exposure times and dose rate may account for the differences in the severity of the effects. Aalapati et al. (2014) observed pulmonary inflammation, necrosis, fibrosis, and granulomas in the lung of mice exposed to 2 mg/m^3^ nano-Ceria for 14 and 28 days resulting in a burden of 1,200 mg/g lung after exposure for 28 days. Rats exposed for 4 weeks in the study reported here inhaled 12.5-fold higher aerosol concentrations, which yielded a lung burden of around 3,000 µg/lung (equivalent to 2,730 µg/g lung) and resulted in less morphological changes. The results of the 2 mg/m^3^ Ceria inhalation study to mice leave open questions which need to be discussed.

### Systemic effects

Substance-related adverse effects after inhalation exposure to two Ceria in the short-term studies were limited to the lung. Potential systemic genotoxicity was assessed by micronuclei test (MNT). The MNT is a frequently used genotoxicity test on nanomaterials (Oesch and Landsiedel 2012). This in vivo assay is able to demonstrate the potential interaction of nanomaterials with chromosomes or mitotic apparatus of replicating erythroblasts as well as their influence on erythropoiesis in the bone marrow (LeBaron et al. 2013). No genotoxic potential after inhalation of Ceria NM-211 or NM-212 was observed in both short-term studies. Cerium bone marrow content was not determined in our inhalation studies, but a tissue concentration of 6.61 ng/g bone marrow was reported 7 days after intratracheal instillation of 1 mg/kg of neutron-activated Ceria NM-212 (Molina et al. 2014). Ceria inhalation exposure has been reported to result in a wide range of systemic responses ranging from no effects to severe effects in blood or extrapulmonary organs, such as liver or kidneys (Aalapati et al. 2014; Gosens et al. 2013; Srinivas et al. 2011). In the study reported here, 5 days of exposure to 25 mg/m^3^ (retained lung burden of 0.53 mg) resulted in higher absolute and relative neutrophil cell counts in the blood, without an increase in total cell counts. This slight neutrophilia was detected directly after the end of exposure and was no longer present after 3 weeks of post-exposure. Considering the pronounced inflammation in the lung at 25 mg/m^3^, the neutrophilia in blood was considered to be secondary to the local effects (systemic acute-phase response). The inflammatory response in the lung based on the increase in neutrophil counts in BALF was also lower after 4 weeks compared to 5 days of exposure. And no altered blood parameters could be detected after 4 weeks of inhalation exposure. Furthermore, a whole panel of extrapulmonary organs and tissues was examined histologically as required by OECD test guideline 412. Very low Cerium contents were detected in the liver at two time points after 4 weeks of inhalation exposure to 25 mg/m^3^ Ceria NM-212 (which is a general finding for inhaled nanoparticles) without any related morphological abnormalities. None of the other extrapulmonary organs showed any morphological abnormalities. The absence of systemic effects is consistent with the very low extrapulmonary tissue Ceria concentrations in this and other studies (Gosens et al. 2013; Geraets et al. 2012; Molina et al. 2014). After instillation of 1 mg/kg body weight neutron-activated ^141^Cer NM-212, only 0.3 % of the administered dose was found in the liver after 28 days post-instillation (Molina et al. 2014). However, after a single intravenous (i.v.) administration of 30 nm nano-Ceria (87 mg/kg body weight) into rats, oxidative stress was seen in liver and spleen and granulomas were observed in the liver (Yokel et al. 2012). The Ceria-induced adverse effects were explained by mechanical irritation or its chemical (catalytic) activity (Yokel et al. 2012). The high Cerium concentrations in the liver after i.v. administration were far beyond the concentrations achievable by oral, dermal, or inhalation exposure. In contrast, severe systemic findings were reported in mice after 28-day inhalation exposure to 2 mg/m^3^ nano-Ceria plus 28 days of post-exposure (lung burden of 500 mg/g tissue). The findings comprised necrosis in kidneys, hepatocytomegaly, and cytoplasmatic vacuolization in the heart (Aalapati et al. 2014), which was claimed to be associated with moderate accumulation of Cerium in extrapulmonary organs. However, even i.v. injection of high doses of Ceria did not lead to similar changes in rat kidney and heart (Yokel et al. 2012).

### Comparison of NM-211 with NM-212

The effects of two different Ceria (NM-211 and NM-212) were compared after 5 days of inhalation exposure. Ceria NM-211 and NM-212 have identical composition, agglomeration state, crystallinity, and insolubility. Compared to Ceria NM-212, NM-211 has considerably smaller primary particles, larger specific surface, significantly fewer organic contaminations on the surface, and reduced photocatalytic activity. Despite these differences, Ceria NM-211 and NM-212 share the same tendency to recrystallize in acidic PSF, which simulates the lysosome of macrophages. These properties are comparable to each other, but significantly different from other metal oxide nanomaterials and seem to be primarily substance-related themselves. Compared to Ceria NM-212, Ceria NM-211 elicited higher increases in lymphocytes, cell mediators (e.g., MCP-1), and neutrophils in BALF at 25 mg/m^3^ with a slower recovery during the post-exposure period. Moreover, significant increase in lung weights was noted in animals exposed to 25 mg/m^3^ Ceria NM-211, but not NM-212. When biological effects (e.g., inflammation) of two particles are compared, the lung burden may be more appropriately expressed as particle volume, particle surface area (Oberdorster et al. 1994b). Morrow first introduced volume as dose metric for overload (Morrow 1988). Pauluhn modified the overload hypothesis by introducing agglomerate density (instead of physical density) into the calculation of volumetric loading (Pauluhn 2011). The effective particle density is needed to calculate the particle volume. In a guidance document published by Webb (2001), various definitions of the powder volumes are given (e.g., apparent density and tapping density). In the current study, an agglomerate density of 2 g/cm^3^ for NM-212 and 0.6 g/cm^3^ for NM-211 was selected. The densities were extracted by integrating the porosity below 1 µm (from Hg intrusion porosimetry) and then derived empirically by approaching MPPD model calculations together with measured mass lung burden. According to our calculation, volumetric lung overload can only be assumed after 4 weeks exposure to 25 mg/m^3^. Impaired lung clearance—which is one of the consequences of lung overload conditions—was, however, already observed after inhalation of 5 mg/m^3^ Ceria.

It is not fully understood which characteristics of nano-Ceria lead to the effects observed in previous and current studies [Environmental Protection Agency (EPA) 2009]. Assessing the immune toxicity of another member of the group of poorly soluble particle namely Titania after instillation in rats, lower phagocytic ability and disruption of pulmonary alveolar macrophage function were correlated with surface reactivity and size of surface area (Liu et al. 2010). A number of studies over the past 15 years suggested that the smaller the particle size (the greater the surface area dose), the greater the induced inflammatory response (Oberdorster et al. 1994a; Cullen et al. 2000; Sager and Castranova 2009; Sager et al. 2008). Furthermore, surface area of retained particles seems to correlate with acute inflammatory reactions after inhalation. The correlation of lung burden, expressed as mass, volume, and surface area, and neutrophil (PMN) counts in BALF as parameter for biological effects is presented in Fig. 7 (Table S9).

In the present study with 5 days of exposure, the larger specific surface area of Ceria NM-211 seems to contribute to the higher biological activity as compared to Ceria NM-212. Surface area normalized the dose response of the two Ceria materials. This result is consistent with other studies, which used surface area as dose metric to quantify the toxicity of the PSPs Titania and barium sulfate (Tran et al. 2000; Oberdorster et al. 1994a, b).

### Comparison of 5 days and 4 weeks of exposure

Rats were exposed to Ceria NM-212 for 5 days and 4 weeks. Exposure to 25 mg/m^3^ for 5 days resulted in the same lung burden as 4 weeks of exposure to 5 mg/m^3^. In consideration of the same lung burdens, the dose rates are 106 µg (5 days of exposure to 25 mg/m^3^) and 26 µg/day (4 weeks of exposure to 5 mg/m^3^) (see Table 5). One should note that the calculated dose rates still include the clearance and, therefore, reflect rather the retained than the deposited Ceria concentration in the lung.

**Table 5:** Dose rate of Ceria NM-212

Study	Test substance	Concentration (mg/m^3^)	Lung burden^a^ (µg)	Dose rate (µg/day)
5 days (5 exposures)	Ceria NM-212	0.5	11	2.2
		5	100	20
		25	530	106
4 weeks (20 exposures)	Ceria NM-212	0.5	41	2.05
		5	520	26
		25	2,620	131

^a^Lung burden values after 5 days of exposure (5 days exposure) and 2 days after the end of exposure (4 weeks exposure)

**Fig. 8:**
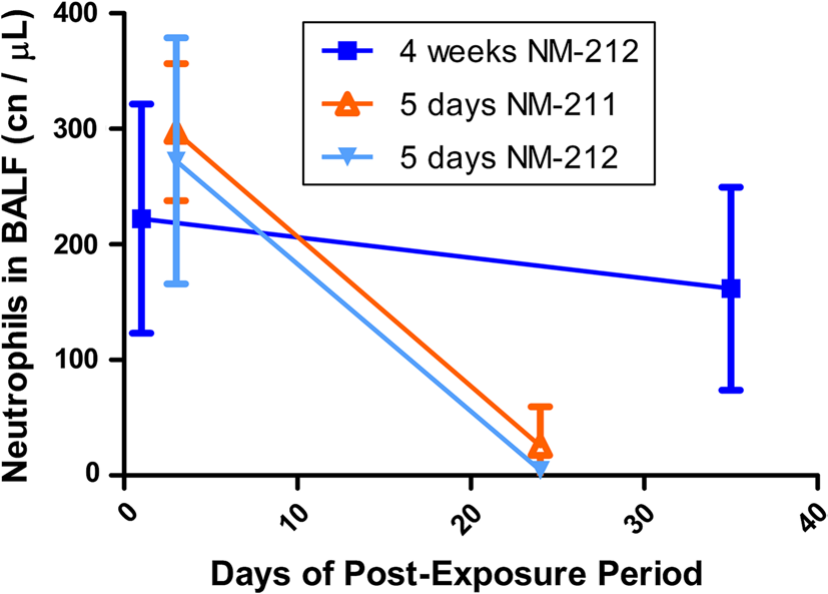
Inflammatory potency of the short-term studies with 5 days and 4 weeks of exposure over post-exposure period: absolute neutrophil counts in BALF in the short-term studies: 3 and 24 days after end of exposure to 25 mg/m^3^ Ceria NM-211 (*orange* 5-day exposure) and NM-212 (*light blue* 5-day exposure) or one and 35 days after the end of exposure to 25 mg/m^3^ NM-212 (*dark blue* 4-week exposure)

**Fig. 9:**
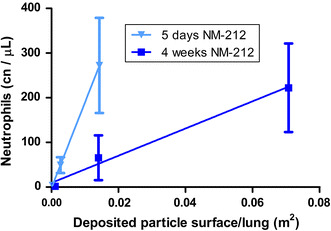
Neutrophil counts in BALF correlated with deposited particle per surface lung after 5-day and 4-week exposure using the aerosol concentrations of 0.5, 5, and 25 mg/m^3^ CeO_2_ NM-212 for each point

The dose rate of particles seems to influence the acute inflammatory reaction in the lung (Baisch et al. 2014). Despite the same lung burdens but different dose rates, a stronger neutrophil response in BALF was observed after the shorter exposure time (Figs. 8, 9). The decay in neutrophil numbers after 4 weeks was by far slower than after 5 days, suggesting that inflammation developing at lower dose rate is longer lasting and more persistent. Moreover, BALF effects almost completely regressed after the end of the 5 days exposure to 5 mg/m^3^ even though the lung burden was only cleared by about 10 % (Fig. 9).

Hence, the dose rate rather than the lung burden seems to drive the neutrophil count in BALF (Fig. 9; Table 5).

Neutrophils are one of the first cell types recruited by inflammatory signals evoked by deposited particles in the lung, with peak numbers within 48 h (Alber et al. 2012). Neutrophils in BALF are a sensitive measure of this initial phase. With sustained inflammatory conditions, neutrophil recruitment undergoes a certain physiological adaptation in the body. The number of neutrophils observed in BALF was decreasing and supplemented by mononuclear cells, especially macrophages that were visible in histopathology but not in BALF. The sustained presence of particles in the lung can drive macrophages to form granulomatous lesions. Although a small dose rate over a long exposure time achieved the same lung burden as a high dose rate over a short time period, the effects observed may be more severe due to consistent irritation of the tissue by small dose rates of fresh materials. Apparently, the initial neutrophil reaction is directed by the dose rate while the progression of the inflammation in the lung is also driven by the continuing presence of particles in the lung.

## Conclusion

**Fig. 10:**
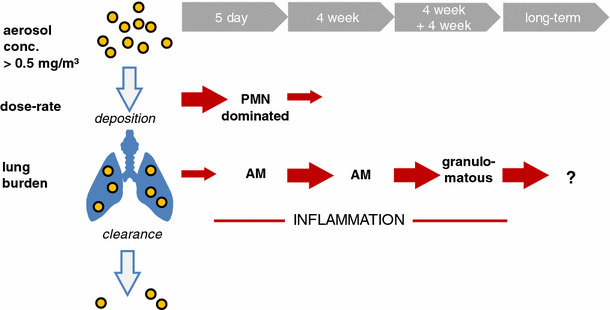
Summary of results after 5 days and 4 weeks of exposure to Ceria

Inhaled nano-Ceria was deposited in the lung. The particles were cleared from the lung with a retention half-time of 40 days at a concentration of 0.5 mg/m^3^; higher aerosol concentrations impaired this clearance. A smaller fraction of the particles was transferred to the lung-associated lymph nodes. The initial inflammatory reaction was observed by an increase in neutrophils which correlated with dose rate rather than absolute lung burden. With time, the neutrophil number decreased. The decrease was observed after the exposure ended but also with sustained exposure (longer than 5 days in the current studies). In the later phase (after 4 weeks of exposure), the inflammatory reaction was dominated by mononuclear cells, especially macrophages. The inflammatory response was driven by the surface of the particles presented to the lung, as this was the dose metrics with the best correlation of the two Ceria materials. An aerosol concentration of 0.5 mg/m^3^ did not cause inflammatory responses in the lung. 5 mg/m^3^ was the lowest aerosol concentration at which the early as well as the later inflammatory response was observed, even though lung burdens were different at the onset of the two phases. The progression of the later inflammatory reaction toward a granulomatous type depended on the duration and amount of the particle (surface) burden in the lung (Fig. 10). Thus, the potency of an inhaled particle to induce inflammatory responses in the lung could be assessed by the aerosol concentration causing the initial neutrophil response, whereas the later granulomatous phase could only be detected by histopathology. The further progression of the biological response was determined by the continuing presence of the particles in the lung. Ultimately, the further progression of the biological responses needs to be studied by the long-term inhalation study assessing the effects as well as the time course of the lung burdens. The results of the short-term studies presented here were used to set the aerosol concentrations of the long-term study with Ceria which is currently being performed. Four aerosol concentrations are being tested, 0.1, 0.3, 1, and 3 mg/m^3^, expected to result in no inflammation and no overload (two concentrations), inflammation without overload, and inflammation and overload, respectively.

## Electronic supplementary material

Below is the link to the electronic supplementary material.

Supplementary material 1 (DOC 796 kb)

## Abbreviations

AAALAC: Association for Assessment and Accreditation of Laboratory Animal Care International
AAN: Average agglomeration numbers
ALP: Alkaline phosphatase
AUC: Area under the curve
BALF: Bronchoalveolar lavage fluid
BALT: Bronchus-associated lymphoid tissue
BET: Brunauer–Emmet–Teller
Ceria: Cerium dioxide
CINC: Cytokine-induced neutrophil chemoattractant
FaSSIF: Fasted state simulated intestinal fluid
GGT: γ-Glutamyltransferase
GSD: Geometric standard deviation
ICP-AES: Inductively coupled plasma atomic emission spectrometry
ICP-MS: Inductively coupled plasma optical emission spectrometry
IL: Interleukin
LDH: Lactate dehydrogenase
M-CSF: Macrophage colony-stimulating factor
MCP: Monocyte chemoattractant protein
MMAD: Mass median aerodynamic diameter
MPPD: Multiple-path particle dosimetry model
NAG: *N*-acetyl-β-glucosaminidase
NOAEC: No observed adverse effect concentration
OPN: Osteopontin
PMN: Polymorphnuclear neutrophil
PSF: Phagolysosomal simulant fluid
PSP: Poorly soluble particles with low toxicity
SAD: Selected area electron diffraction
SEM: Scanning electron microscopy
SIMS: Secondary ion mass spectrometry
SMPS: Scanning mobility particle sizer
STIS: Short-term inhalation study protocol
TEM: Transmission electron microscopy
TGA: Thermogravimetric analysis
TiO_2_: Titanium dioxide
XPS: X-ray photoelectron spectroscopy
