# Supplementary material for: Time course of lung retention and toxicity of inhaled particles: short-term exposure to nano-Ceria
Source: Arch Toxicol. 2014 Oct 2;88(11):2033–59. doi: 10.1007/s00204-014-1349-9 (PMC4555363; doi:10.1007/s00204-014-1349-9)
Supplement: Supplementary file 1 — Supplementary material 1 (DOC 796 kb) [file 204_2014_1349_MOESM1_ESM.doc]

Supplementary Information


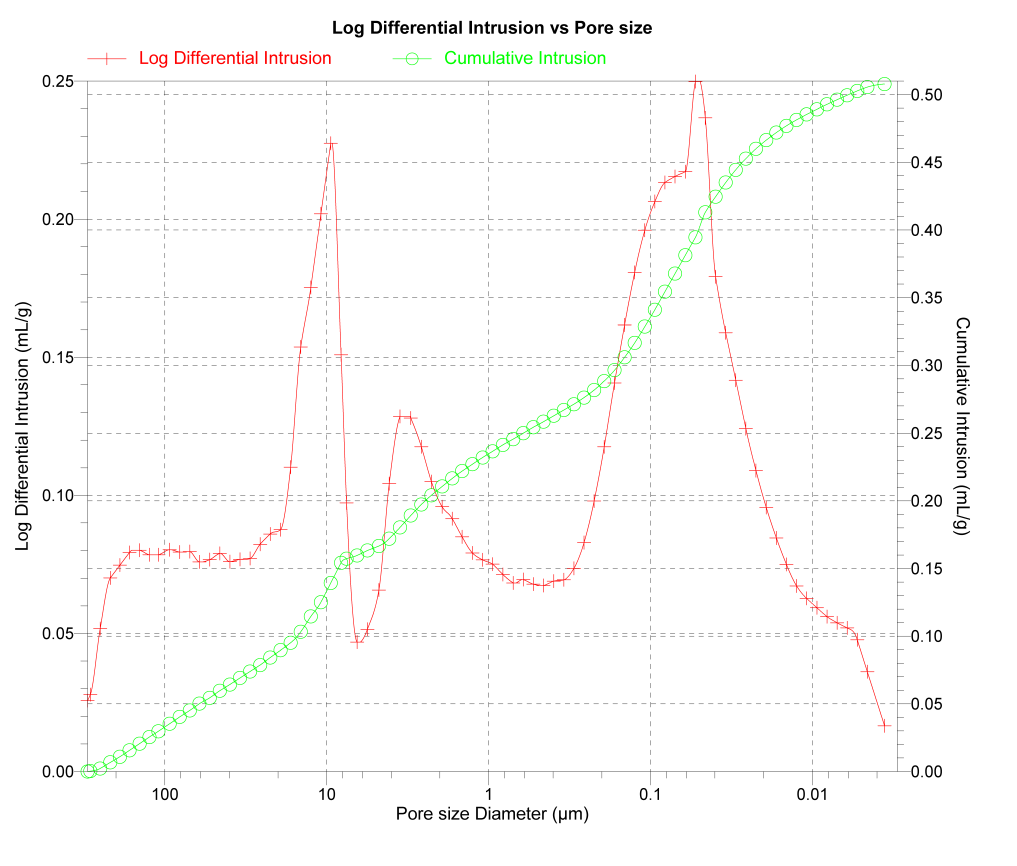


A


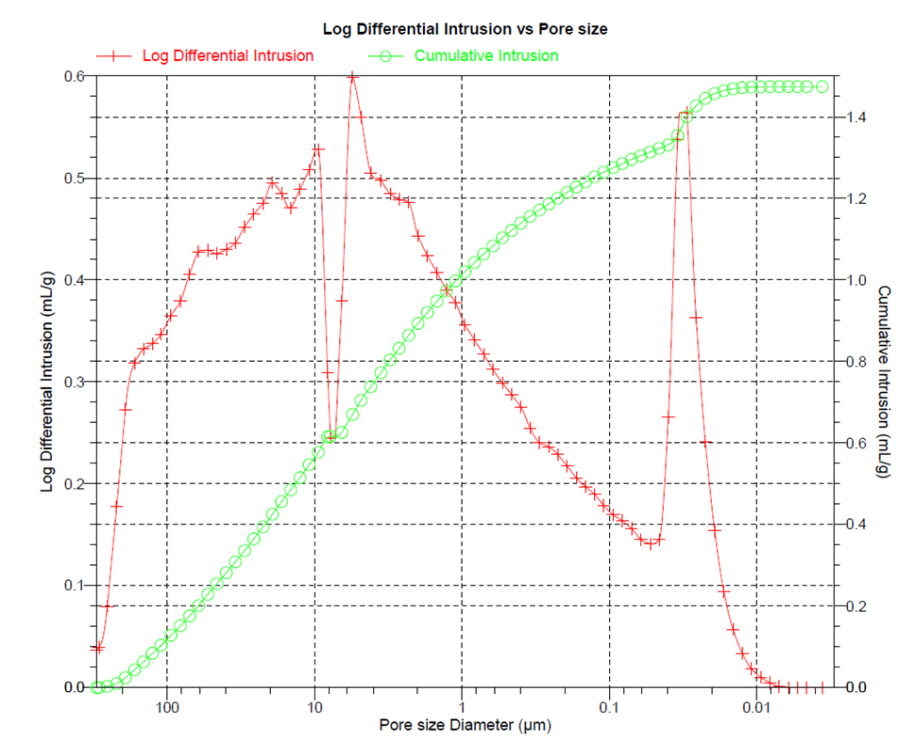


B

Fig. S1 Diagrams of mercury (Hg) intrusion porosimetry experiments with Ceria

A NM-211 and B NM-212

Tab. S2 Mean clinical pathology parameters in blood after exposure to Ceria

|  | **5 days of exposure** | | | | | | **4 weeks of exposure** | | | |
| --- | --- | --- | --- | --- | --- | --- | --- | --- | --- | --- |
|  | **Control** | **NM-212** | | | **NM-211** | | **Control** | **NM-212** | | |
| **Target conc. [mg/m^3^]** | **0** | **0.5** | **5** | **25** | **0.5** | **25** | **0** | **0.5** | **5** | **25** |
| Measured conc. (mg/m³)+SD | 0 | 0.48 ± 0.0 | 5.2 ± 1.1 | 25.6 ± 6.0 | 0.45 ± 0.1 | 25.8 ±1.7 | 0 | 0.5 ±0.2 | 5.3 ±0.9 | 25.9 ±6.0 |
| **Blood cells** |  |  |  |  |  |  |  |  |  |  |
| **Neutrophils [giga/L]** +SD |  |  |  |  |  |  |  |  |  |  |
| Time point 1^1^ | 0.55 ± 0.21 | 0.52 ± 0.07 | 0.92* ± 0.22 | 1.08** ± 0.14 | 0.67 ± 0.16 | 1.14* ± 0.30 | 1.19 ± 0.95 | 0.73 ± 0.24 | 0.83 ± 0.33 | 1.18 ± 0.32 |
| Time point 2^2^ | 0.64 ± 0.19 | 0.58 ± 0.02 | 0.59 ± 0.13 | 0.64 ± 0.10 | 0.73 ± 0.26 | 0.75 ± 0.23 | 0.72 ± 0.26 | 0.71 ± 0.24 | 0.85 ± 0.17 | 1.20 ± 0.37 |
| **Lymphocytes [giga/L]** +SD |  |  |  |  |  |  |  |  |  |  |
| Time point 1^1^ | 3.16 ± 1.09 | 2.55 ± 0.72 | 3.77 ± 1.16 | 2.90 ± 0.56 | 3.12 ± 0.85 | 3.34 ± 1.09 | 3.67 ± 1.13 | 3.47 ± 0.72 | 3.01 ± 0.53 | 2.65 ± 0.62 |
| Time point 2^2^ | 3.00 ± 0.66 | 3.12 ± 1.27 | 4.85** ± 0.57 | 3.15 ± 0.51 | 2.24 ± 0.39 | 3.19 ± 0.40 | 2.86 ± 0.73 | 3.65 ± 1.14 | 2.95 ± 0.88 | 3.26 ± 0.63 |
| **Neutrophils [%]** +SD |  |  |  |  |  |  |  |  |  |  |
| Time point 1^1^ | 14.1 ± 0.9 | 17.0 ± 5.3 | 19.9 ± 8.0 | 26.2** ± 4.5 | 17.4 ± 5.0 | 25.0** ± 7.8 | 22.2 ± 14.5 | 16.4 ± 3.8 | 20.0 ± 4.4 | 29.9 ± 6.2 |
| Time point 2^2^ | 17.3 ± 6.8 | 15.3 ± 6.4 | 10.4** ± 1.9 | 16.5 ± 3.8 | 22.1 ± 7.4 | 18.3 ± 6.0 | 19.4 ± 7.8 | 15.9 ± 5.2 | 21.8 ± 3.6 | 25.6 ± 3.9 |
| **Lymphocytes [%]** +SD |  |  |  |  |  |  |  |  |  |  |
| Time point 1^1^ | 81.6 ± 1.4 | 78.4 ± 5.5 | 76.0 ± 8.5 | 69.3** ± 5.7 | 77.6 ± 4.7 | 70.5* ± 8.3 | 72.3 ± 14.2 | 78.2 ± 4.3 | 75.2 ± 5.5 | 66.1 ± 5.8 |
| Time point 2^2^ | 78.3 ± 7.1 | 80.5 ± 7.9 | 85.6 ± 2.4 | 79.7 ± 4.4 | 72.9 ± 7.6 | 78.1 ± 5.9 | 75.7 ± 8.6 | 79.3 ± 6.6 | 73.4 ± 3.6 | 70.2 ± 4.0 |

* statistically significant, p <= 0.05; ** statistically significant, p <= 0.01.
^1^ time point 1 is three (5 days of exposure) or two days (4 weeks of exposure) after the end of exposure; ^2^ time point 2 is 24 (5 days of exposure) or 35 days (4 weeks of exposure) after the end of exposure

SD: standard deviation

A

B

## Tab. S3 Systemic Genotoxicity (Micronucleus Test): means of all test groups (5 individuals per group) after 5 days and 4 weeks of exposure to Ceria

| **Study** | **Concentration** | **Parameter** | **%RET** | **%MN-RET** | **%MN-NCE** |
| --- | --- | --- | --- | --- | --- |
| 5 days of exposure  Three days after the end of exposure | control | No. RET mean | 0.68 | 0.085 | 0.007 |
|  | 0.5 mg/m³ NM-212 | No. RET mean | 1.23 | 0.092 | 0.005 |
|  | 5 mg/m³ NM-212 | No. RET mean | 1.19 | 0.095 | 0.013 |
|  | 25 mg/m³ NM-212 | No. RET mean | 1.05 | 0.070 | 0.010 |
|  | 0.5 mg/m³ NM-211 | No. RET mean | 0.89 | 0.084 | 0.008 |
|  | 25 mg/m³ NM-211 | No. RET mean | 0.83 | 0.070 | 0.010 |
|  | negative control | No. RET mean | 6.10 | 0.21 | 0.021 |
|  | positive control | No. RET mean | 1.01 | 1.37 | 0.029 |
| 4 weeks of exposure  Two days after the end of exposure | control | No. RET mean | 1.01 | 0.049 | 0.005 |
|  | 0.5 mg/m³ NM-212 | No. RET mean | 1.07 | 0.079 | 0.007 |
|  | 5 mg/m³ NM-212 | No. RET mean | 1.11 | 0.065 | 0.008 |
|  | 25 mg/m³ NM-212 | No. RET mean | 0.98 | 0.076 | 0.007 |
|  | negative control | No. RET mean | 6.26 | 0.16 | 0.022 |
|  | positive control | No. RET mean | 1.04 | 1.77 | 0.031 |

RET: reticulocyte; MN-RET: micronucleated reticulocyte; MN-NCE: normochromatic erythrocytes

Tab. S4 Historical control ranges of parameters in bronchoalveolar lavage fluids of female Crl:Wi(Han) rats aged 7 to 12 weeks. Means of 47 sampling dates (5 individuals per group) as well as the minimum and maximum mean of all studies are mentioned (exposure period 1 to 4 weeks).

| **Parameter** | **Unit** | **Mean** | **Minimum** | **Maximum** |
| --- | --- | --- | --- | --- |
| Absolute neutrophil counts | cn/µL | 0.54 | 0.07 | 2.80 |
| Alkaline phosphatase (ALP) | µkat/L | 0.52 | 0.23 | 0.87 |

Tab. S5 Summary of pathological findings after exposure to Ceria

|  | **5 days of exposure to NM-212** | | | | | | | **5 days of exposure to NM-211** | | | | | | **4 weeks of exposure to NM-212** | | | | | | | | | | |
| --- | --- | --- | --- | --- | --- | --- | --- | --- | --- | --- | --- | --- | --- | --- | --- | --- | --- | --- | --- | --- | --- | --- | --- | --- |
|  | After 5 days of exposure | | | 21 days after the end of exposure | | | | After 5 days of exposure | | 21 days after the end of exposure | | | | Two days after the end of exposure | | | | | 34 days after the end of exposure | | | | | |
| **Target conc. [mg/m^3^]** | **0.5** | **5** | **0.5** | **5** | **0.5** | | **5** | **0.5** | **25** | **0.5** | | **25** | | **0.5** | | **5** | **25** | | **0.5** | | **5** | | **25** | |
| **Mean lung weights (%)**  compared to the control group (set to 100%) |  | | | | | | |  | | | | | |  | | | | | | | | | | |
| **Absolute** | - | - | - | - | | - | - | 93 | **124**** | | - | | - | 102 | | **113**** | | **130*** | | 100 | | 105 | | **116**** |
| **Relative** to body weight | - | - | - | - | | - | - | 96 | **120**** | | - | | - | 99 | | **110**** | | **129**** | | 102 | | 104 | | **120*** |
| **Macroscopic findings**  (number of affected animals) |  | | | | | | |  | | | | | |  | | | | | | | | | | |
| **Mediastinal lymph nodes enlarged** | **1** | **2** | **2** | - | | **1** | **1** | **3** | **4** | | **1** | | **2** | - | - | | | **2** | - | | **2** | | **8** | |

*p<0.05, **p<0.01

Tab. S6 Lung burden analysis

A

| 5 days of exposure | | Lung Burden [mg] ± SD | |
| --- | --- | --- | --- |
| Test substance | Conc.  [mg/m³] | After 5 days of exposure | 21 days after the end of exposure |
| NM-212 | 0.5 | 0.011  ±0.001 | 0.006  ±0.001 |
|  | 5 | 0.1 ±0.009 | 0.088  ±0.009 |
|  | 25 | 0.53  ±0.12 | 0.4  ±0.07 |
| NM-211 | 0.5 | 0.006 ±0.001 | 0.0038  ±0.0004 |
|  | 25 | 0.28  ±0.02 | 0.26  ±0.04 |

B

| 4 weeks of exposure | | Lung Burden [mg] ± SD | | | | | | |
| --- | --- | --- | --- | --- | --- | --- | --- | --- |
| Test substance | Conc.  [mg/m³] | One day after the end of exposure | Two days after the end of exposure* | Three days after the end of exposure | Nine days after the end of exposure | 35 days after the end of exposure | 65 days after the end of exposure | 129 days after the end of exposure |
| NM-212 | 0.5 | 0.041  ±0.007 | 0.055  ±0.01 | 0.039  ±0.005 | 0.040  ±0.002 | 0.023  ±0.004 | 0.017  ±0.01 | 0.009  ±0.0037 |
|  | 5 | 0.52 ±0.06 | 0.59  ±0.13 | nd | nd | 0.56 ±0.05 | nd | nd |
|  | 25 | 2.62  ±0.29 | 3.14  ±0.5 | 2.21  ±0.12 | 2.54  ±0.07 | 2.47  ±0.18 | 2.46  ±0.13 | 1.8  ±0.26 |

nd: not determined; SD: standard deviation

* Two days after the end of exposure, the measured mean lung burdens were higher than on the previous. These values were calculated from lung burdens of the left half lungs of five animals per test group, whereas lung burdens at all other time points were measured using the entire lungs. These data were disregarded for half-time calculations.

Tab. S7 Burden analysis of lung associated lymph nodes

A

| 5 days of exposure | | Lymph nodes burden [µg] ± SD | |
| --- | --- | --- | --- |
| Test substance | Conc.  [mg/m³] | After 5 days of exposure | 21 days after the end of exposure |
| NM-212 | 25** | 1.7  ±0.6 | 5  ±4 |
| NM-211 | 25** | 1.4  ±0.6 | 3  ±0.1 |

**Values of 0.5 mg/m³, 5 mg/m³ CeO_2_ NM-212 and 0.5 mg/m³ Ceria NM-211 were below the analytical limit of quantification (LOQ);
SD: standard deviation

B

| 4 weeks of exposure | | Lymph nodes burden [µg] ± SD | | | |
| --- | --- | --- | --- | --- | --- |
| Test substance | Conc.  [mg/m³] | Three days after the end of exposure | Nine days after the end of exposure | 65 days after the end of exposure | 129 days after the end of exposure |
| NM-212 | 25* | 10  ±20 | 10  ±10 | 210  ±30 | 350  ±70 |

*Values of 0.5 mg/m³ and 5 mg/m³ Ceria NM-212 were below the analytical limit of quantification (LOQ); SD: standard deviation

Tab. S8 Burden analysis of liver

| 4 weeks of exposure | | Liver burden [µg] ± SD | |
| --- | --- | --- | --- |
| Test substance | Conc.  [mg/m³] | Three days after the end of exposure | 65 days after the end of exposure |
| NM-212 | 25 | 1.56 ± 1.32 | 1.93 ± 0.91 |

SD: standard deviation

Tab. S9 Lung burden expressed by different dose-metrics

|  |  | | Lung Burden | | |
| --- | --- | --- | --- | --- | --- |
| Study | Test substance | Conc.  [mg/m³] | Mass  [mg] ± SD | Surface area  [m²] | Volume [µL] |
| 5 days  of exposure | NM-212 | 0.5 | 0.011  ±0.001 | 0.00027 | 0.005 |
|  | NM-212 | 5 | 0.1 ±0.009 | 0.00270 | 0.05 |
|  | NM-212 | 25 | 0.53  ±0.12 | 0.01431 | 0.27 |
| 4 weeks  of exposure | NM-212 | 0.5 | 0.041  ±0.007 | 0.00108 | 0.02 |
|  | NM-212 | 5 | 0.52 ±0.06 | 0.01404 | 0.26 |
|  | NM-212 | 25 | 2.62  ±0.29 | 0.07074 | 1.31 |
